# KLF17 is an important regulatory component of the transcriptomic response of Atlantic salmon macrophages to *Piscirickettsia salmonis* infection

**DOI:** 10.3389/fimmu.2023.1264599

**Published:** 2023-12-14

**Authors:** Diego Pérez-Stuardo, Mateus Frazão, Valentina Ibaceta, Bernardo Brianson, Evelyn Sánchez, J. Andrés Rivas-Pardo, Eva Vallejos-Vidal, Felipe E. Reyes-López, Daniela Toro-Ascuy, Elena A. Vidal, Sebastián Reyes-Cerpa

**Affiliations:** ^1^ Centro de Genómica y Bioinformática, Facultad de Ciencias, Ingeniería y Tecnología, Universidad Mayor, Santiago, Chile; ^2^ Programa de Doctorado en Genómica Integrativa, Vicerrectoría de Investigación, Universidad Mayor, Santiago, Chile; ^3^ Escuela de Biotecnología, Facultad de Ciencias, Ingeniería y Tecnología, Universidad Mayor, Santiago, Chile; ^4^ Agencia Nacional de Investigación y Desarrollo (ANID) Millennium Science Initiative Program-Millennium Institute for Integrative Biology (iBio), Santiago, Chile; ^5^ Núcleo de Investigaciones Aplicadas en Ciencias Veterinarias y Agronómicas, Facultad de Medicina Veterinaria y Agronomía, Universidad De Las Américas, La Florida, Santiago, Chile; ^6^ Centro de Biotecnología Acuícola, Facultad de Química y Biología, Universidad de Santiago de Chile, Santiago, Chile; ^7^ Centro de Nanociencia y Nanotecnología (CEDENNA), Universidad de Santiago de Chile, Santiago, Chile; ^8^ Laboratorio de Virología, Departamento de Biología, Facultad de Ciencias, Universidad de Chile, Santiago, Chile

**Keywords:** *Piscirickettsia salmonis*, host-pathogen interaction, macrophage polarization, gene regulatory network, Atlantic salmon

## Abstract

*Piscirickettsia salmonis* is the most important health problem facing Chilean Aquaculture. Previous reports suggest that *P. salmonis* can survive in salmonid macrophages by interfering with the host immune response. However, the relevant aspects of the molecular pathogenesis of *P. salmonis* have been poorly characterized. In this work, we evaluated the transcriptomic changes in macrophage-like cell line SHK-1 infected with *P. salmonis* at 24- and 48-hours post-infection (hpi) and generated network models of the macrophage response to the infection using co-expression analysis and regulatory transcription factor-target gene information. Transcriptomic analysis showed that 635 genes were differentially expressed after 24- and/or 48-hpi. The pattern of expression of these genes was analyzed by weighted co-expression network analysis (WGCNA), which classified genes into 4 modules of expression, comprising early responses to the bacterium. Induced genes included genes involved in metabolism and cell differentiation, intracellular transportation, and cytoskeleton reorganization, while repressed genes included genes involved in extracellular matrix organization and RNA metabolism. To understand how these expression changes are orchestrated and to pinpoint relevant transcription factors (TFs) controlling the response, we established a curated database of TF-target gene regulatory interactions in *Salmo salar*, SalSaDB. Using this resource, together with co-expression module data, we generated infection context-specific networks that were analyzed to determine highly connected TF nodes. We found that the most connected TF of the 24- and 48-hpi response networks is KLF17, an ortholog of the KLF4 TF involved in the polarization of macrophages to an M2-phenotype in mammals. Interestingly, while KLF17 is induced by *P. salmonis* infection, other TFs, such as NOTCH3 and NFATC1, whose orthologs in mammals are related to M1-like macrophages, are repressed. In sum, our results suggest the induction of early regulatory events associated with an M2-like phenotype of macrophages that drives effectors related to the lysosome, RNA metabolism, cytoskeleton organization, and extracellular matrix remodeling. Moreover, the M1-like response seems delayed in generating an effective response, suggesting a polarization towards M2-like macrophages that allows the survival of *P. salmonis*. This work also contributes to SalSaDB, a curated database of TF-target gene interactions that is freely available for the Atlantic salmon community.

## 1 Introduction

*Piscirickettsia salmonis* is the etiological agent of Salmonid Rickettsial Septicaemia (SRS), a contagious systemic disease mainly affecting saltwater salmon (1). *P. salmonis* is a Gram-negative bacterium described as a facultative intracellular pathogen that resides in vacuoles of macrophages and hepatocytes (1–3). In Chile, the appearance of recurrent and aggressive outbreaks of SRS is the most severe health threat to the salmon industry. In the first half of 2022, mortalities associated with *P. salmonis* represented 54.2% of the total mortalities attributed to infectious causes in Atlantic salmon (*Salmo salar*) (4). The prophylaxis and control against *P. salmonis* have mainly depended on vaccines and antibiotics. However, neither strategy has been effective (5, 6) because vaccines offer short-term protection, and antibiotics are inefficient against intracellular pathogens (5–11).

Despite the severe impact of *P. salmonis* on Chilean aquaculture, its life cycle and pathogenic mechanisms are poorly characterized (12). Upon entry into macrophages through phagocytosis, *P. salmonis* induces a significant cytoskeletal reorganization through actin disorganization (13, 14), where they survive and replicate within vacuoles (14–16). *P. salmonis* evades the lysosomal degradation and inhibits the cell proteolytic activity (12, 14, 15, 17), persists in macrophages (14, 18), modulates apoptosis (14, 16), and inhibits oxidative stress (14, 19–23). Moreover, *P. salmonis* induces the expression of an anti-inflammatory milieu, probably to ensure survival by downregulating the host antimicrobial response (14, 21, 24, 25). Despite the extensive evidence suggesting that macrophages infected by *P. salmonis* see their effector functions affected, the regulatory mechanisms of the immune response modulated during the infection, allowing the pathogen to survive, remain unclear.

Macrophages are essential in initiating the pro-inflammatory response against a pathogen. However, they show adapted effector functions for an immune response, and repair previously generated damage. In mammals, this opposite role is known as macrophage polarization, where macrophages can specialize in two opposite phenotypes, M1 and M2 (26, 27). The M1-like profile is associated with a pro-inflammatory macrophage, associated with the production of T helper-1 (Th1) cells and cytokines, such as IL-12, IL-1β, and TNFα, and antimicrobial molecules, such as nitric oxide (NO) and reactive oxygen species (ROS). Conversely, the M2-like profile is associated with Th2 cells and a milieu enriched in anti-inflammatory cytokines, high expression of arginase, IL-1decoyR, IL-10, and IL-1RA, and high phagocytic activity (26, 28–36).

Macrophage polarization is a process tightly regulated by transcription factors (TFs) and associated with extensive transcriptional reprogramming changes. In mammals, the TFs that regulate polarization are widely studied, while in fish, this area is poorly understood (37). In mammals, several TFs, such as peroxisome proliferator-activated receptors (PPARs), signal transducers and activators of transcription (STATs), CCAAT-enhancer-binding proteins (C/EBPs), interferon regulatory factors (IRFs), Krüppel-like factors (KLFs), GATA binding protein (GATA) 3, nuclear transcription factor-kappa β (NF-kβ), c-MYC, and MafB promote the expression of specific genes, which drive the polarization of the macrophages (30, 38). In teleost fish, macrophage polarization has been described as similar to polarization described in mammals (26, 39–41). For example, M1-like macrophages of carp show increased expression of IL-1β and NO production after stimulation with LPS and IFNγ (26, 39–41). In carp and goldfish macrophages, an M2-like profile with a high arginase activity is induced by cAMP and IL-4 (26, 39, 42). In Atlantic salmon, M2-like macrophages seem to be induced for *Piscine orthoreovirus* 1 (PRV-1) infection due to increased detection of arginase-2 after 4- and 6-week post-challenge, which is associated with a fast recovery following viral clearance (43). Smith et al. characterized the mRNA profile of adherent head kidney leukocytes (HKLs) from Atlantic salmon, which differentiate from monocyte-like cells to macrophage-like cells. The authors observed changes in mRNA expression, including changes in immune-related transcripts (*csfr1, arg1, tnfα, mx2*) and TFs involved in mammal’s macrophage polarization (*klf2, klf9, irf7, irf8, stat1*). Many of the TFs and molecules related to immune response identified are markers of macrophages involved in M1/M2 polarization in other species, suggesting a conserved function for some transcripts. Smith et al. observed that the KLF family was downregulated, and members of the IRF family (*irf3, irf7, and irf8*) and *stat1* were upregulated, suggesting that HKLs differentiate to M1-like macrophages (37).

Although pathogens that interfere with M1-like polarization or induce an M2-like phenotype in infected macrophages have not yet been described in fish, manipulating mammals’ macrophage polarization states is emerging as an important pathogenesis mechanism of intracellular bacteria (44). M2-like macrophages appear as a favorable niche for the long-term persistence of numerous intracellular pathogens, such as *Mycobacteria*, *Salmonella*, *Brucella*, *Leishmania*, *Francisella*, and *Listeria*, which can modulate the STAT6-PPARγ/δ pathways to avoid M1-like macrophages polarization (45, 46). Other pathogens, such as *Mycobacterium* and *Coxiella*, seem to differentially regulate polarization to M1-like or M2-like phenotypes depending on their infection stage (44); both bacteria modulate the polarization to take advantage of pro-inflammatory response to promote a chronic infection associated to M1-like phenotype. Then, induce an M2-like phenotype associated with an anti-inflammatory milieu to promote tissue repair but contribute to the evasion of the immune response (44, 47–49). Although *P. salmonis* infection in Atlantic salmon macrophages has not been associated with an M1/M2 profile, the response developed by the infected macrophages could suggest an M2-like polarization, which could explain the bacterial survival and evasion of the effector response.

The immune response of Atlantic salmon infected by *P. salmonis* has been characterized mainly through the use of transcriptomic analysis in tissue from infected fish (21, 22, 50, 51), highlighting an imbalance response that promotes cell survival and proliferation, decrease the adaptive immune response (22), but activate endocytosis and phagocytosis (52), and modulate the iron and amino acid metabolism, which is convenient for intracellular bacteria that uptake nutrients from its host (21, 50, 51, 53, 54). However, these advances in the understanding are only a global description of the cellular processes modulated in infected fish (21, 22, 50, 51); it is still unknown which modulated master regulators are involved in the macrophage immune response when infected by *P. salmonis.*

Gene regulatory network (GRN) analysis is a powerful tool to study complex processes containing the regulatory interactions between TFs and target genes. GRNs are widely used to understand regulatory cascades in different organisms as the infective mechanisms of bacterial pathogens like *Salmonella, Pseudomonas aeruginosa*, and *Legionella pneumophila* (55–59). GRNs have also been applied to understand the immune response, identifying key regulatory cascades in the analysis of gene expression in septic infections (60), identification of novel TFs linked to the control of stress−related immune response (61), and even include data from single-cell RNA-seq to understand the regulatory cascades for different cellular populations (62). In mammalian macrophages, dissecting GRNs has allowed for modeling the polarization process identifying the main phenotypes and several hybrid phenotypes associated with pathological conditions. In this modeling, hybrid phenotypes mimic a hypothetical continuum of macrophage polarization, with M1 and M2 as the extremes of an uninterrupted sequence of states (63). The GRN has also allowed the identification of two metabolic switches during macrophage polarization, where catabolic processes associated with obtaining energy, such as catabolism for nucleotides, cellular macromolecules, and carbohydrates, were upregulated in M1-like macrophages. Conversely, in M2-like macrophages, the anabolic processes, such as the biosynthesis of amino acids and nucleic acids, fatty acid metabolism, and oxidative phosphorylation were upregulated (64). In M1-like macrophages, this transcriptional reprogramming observed enables a fast energy supply needed for cytokine production and the effective eradication of invading pathogens. In turn, the M2-like macrophages show an upregulation of oxidative phosphorylation and anabolic processes, possibly associated with promoting wound healing processes or supporting the survival of M2-like macrophages during longer and persisting parasitic infections (64).

Our work evaluates how Atlantic salmon macrophages respond to early-stage infection by *P. salmonis* at a global transcriptomic level, focusing on deciphering the GRNs orchestrating this response. To improve our understanding, we manually curated poorly described genes by bibliographic revision of genes related to the immune response and endocytic pathway. As a result, we generated a GRN that shows the interactions between TFs and target genes using publicly available databases. We aimed to identify which regulatory processes of the macrophage polarization are modulated by the infection, which consequently allows the bacteria to take control of macrophages and even reside in intracellular vacuoles. Our goal was to uncover how *P. salmonis* induces an immunosuppressive phenotype in infected macrophages, possibly linked to an anti-inflammatory M2-like profile.

## 2 Methods

### 2.1 Cell cultivation

Our experiments used macrophage-like cells SHK-1 (ECACC 97111106) as macrophage models (65), which have been widely used to evaluate salmonid macrophage host interactions with *P. salmonis* (13, 66–68). These cells were maintained at 16°C in Leibovitz medium (L-15; Corning, New York, USA), supplemented with 36 µM 2−mercaptoethanol (2−ME; Gibco, Thermo Scientific, Massachusetts, USA), 10% fetal bovine serum (FBS) (v/v), and 2.5 mg/mL amphotericin B (Corning). The cells were seeded at 6,000 cells/cm^2^ before further experiments, obtaining 10,000 cells/cm^2^ to start the analysis.

Culture and propagation of *P. salmonis* (LF−89−like) was performed in salmonid cell line CHSE 214 (ATCC N°CRL-1682) as previously described by Fryer et al., 1992 (69) and also used in our previous works (15, 17). The CHSE-214 cell line was maintained at 16°C in minimal essential medium (MEM; Corning) supplemented with 10% (v/v) FBS (Hyclone), 10 mM HEPES buffer (Corning), and 1% (v/v) non-essential amino acids (Corning). The infection was observed through conventional inverted light microscopy (Motic AE31E, Leica Microsystems, Wetzlar, Germany) after 4 to 6 days post-infection (dpi) to determine the cytopathic effect on cells (70). Bacteria were quantified using a Petroff−Hausser chamber (Hausser Scientific, Pennsylvania, USA) according to the instructions provided by the manufacturer.

### 2.2 Infection

To obtain the transcriptomic profiles of SHK-1 macrophages-like cells infected by *P. salmonis* at the early stages of infection, we evaluated the response of SHK-1 cells infected by *P. salmonis* at two different time points, 24- and 48-hours post-infection (hpi). We defined an early stage of infection as the time before the infected cells show the appearance of cytopathic effect (CPE), a characteristic event in the progression of the infection by *P. salmonis* in Atlantic salmon macrophage-like cell lines that appears from 72-hpi in advance (19, 71), and therefore, within this time, the key events for infection establishment occur.

The infection was performed using a multiplicity of infection (MOI) of 50 bacteria per cell. SHK-1 cells were washed thrice and then incubated with *P. salmonis* in a minimal medium volume, centrifuged at 100 g for 3 minutes, and then incubated for 1 hour. After the incubation, we added medium to complete the volume recommended for cell cultivation maintenance; in our case, as we used T-75 cultivation bottles, we completed a final volume of 16 mL. As a control, we analyzed non-infected macrophages. We performed all the experiments in 4 replicates.

### 2.3 RNA extraction and sequencing

The RNA was isolated using the mirVana kit following the manufacturer’s instructions. Briefly, the cell cultures were washed thrice with PBS to eliminate cellular debris and medium culture traces. The cells were treated with the disruption solution from the kit and then transferred to the affinity columns provided by the manufacturer to eliminate contaminants and finally elute the purified RNA in a clean tube. The purified RNA was quantified using a fluorometric assay in a Qubit 2.0 following the manufacturer’s instructions with the RNA BR assay kit (Thermofisher).

For sequencing, we used a Custom AnyDeplete kit (Tecan), which we designed to carry out the depletion of Atlantic salmon rRNA (**Supplementary File 1**). Library preparation was performed with Universal plus RNA-seq library preparation kit with NuQuant (Tecan) following the instructions provided by the manufacturer. Library quantity and quality were assessed by Qubit 2.0 (Thermofisher) and TapeStation D1000 ScreenTape (Agilent Technologies Inc.), respectively. The libraries obtained have 350 bp with an insert of 200 bp, including the Illumina 8−nt unique dual indices. Sequencing was performed in an Illumina NovaSeq S4 device, with a configuration of 150 pair-end for 50 million pair-end reads per sample.

### 2.4 Data analysis

RNA-seq read quality was analyzed using FastQC v.0.12.1 (72). Possible adaptor sequences were removed using BBDuk from the BBTools toolkit (https://jgi.doe.gov/data-and-tools/software-tools/bbtools/), using a list of Illumina adaptors specified in **Supplementary File 2**. To visualize the quality of the sequencing files, we used MultiQC v.1.13 (73).

To perform a pseudo-alignment of our reads against the transcriptome of Atlantic salmon, we used Kallisto v.0.46.1 (74). The transcriptome was obtained from ENSEMBL, and we used the most recent version to date (3.1). The transcript-to-gene table needed to collapse the quantifications of the different transcripts into one gene was obtained using the R package BioMart (75, 76). To quantify the aligned reads and perform the statistical analysis to obtain the differentially expressed genes (DEGs), we used DESeq2 (77). The statistical analysis was performed by comparing both times analyzed individually against the non-infected control (24-hpi vs non-infected; 48-hpi vs non-infected). We filtered out the genes that presented less than 10 assigned reads and filtered our DEGs by an adjusted p-value of < 0.1. We did not filter by any value of Fold-change, as we aim to identify novel TFs, and in that context, the slightest change in the gene expression of a TF could mean a complete change in the global gene expression.

The comparison between the obtained in this work and DEGs obtained in different previous studies was performed using GeneSectR (https://github.com/NateyJay/genesectR), a tool based on a one-tailed Fisher Exact Test measuring the probability that two gene lists overlap by more than would be expected by chance (based on the GeneSect tool available in the VirtualPlant 1.3 platform) (78, 79). We used gene lists of DEGs of previous studies from Atlantic salmon infected by *P. salmonis* and other pathogens to compare our data obtained by RNA-seq to determine the association grade with those datasets and the probability of the interception of the given gene lists. We took into consideration gene lists from transcriptomic studies that analyzed Atlantic salmon infected by *P. salmonis*, obtaining three datasets that analyzed different tissues from infected fish, such as liver, spleen, muscle, and head-kidney (27, 50, 80); we also selected studies performed in SHK-1 macrophage-like cell challenged with virus to determine if the response that we observed was similar to any of those conditions (81, 82). As an unrelated dataset, we selected a study that performed RNA-seq analysis on intestinal tissue from Atlantic salmon fed with different diets (83).

In order to identify expression patterns of the DEGs, we performed a weighted co-expression analysis using the WGCNA package (84, 85). Normalized expression of DEGs was used as input for WGCNA. A soft threshold power of 27 was chosen to construct the adjacency matrix, considering a scale-free topology model fit R^2^ of 0.7 and an average connectivity of 10. Network modules were identified using dynamic tree cut, using a minimum cluster size of 10 and a merging threshold of 0.3. Correlation networks were constructed using the corresponding topology overlap matrix, and a threshold of >=0.15 was considered for edge weights. Functional enrichment analysis was performed using the Cytoscape application ClueGo and CluePedia (86, 87). We used the latest annotation version for Atlantic salmon (2017), using all the Gene Ontology (GO) databases. We used mostly the default settings, modifying the following: p-value < 0.05, Min level = 1, Max level = 20, % Genes = 10%, and GO term fusion selected. Networks were visualized using Cytoscape v.3.9.1 software (https://cytoscape.org) (88).

### 2.5 Database creation: SalSaDB

To analyze the regulatory cascades involved in polarization, differentiation, and function of macrophages of Atlantic salmon, we generated the database SalSaDB by gathering information on *S. salar* gene annotation (gene IDs, gene names, gene descriptions) from NCBI and ENSEMBL and regulatory TF-target gene data from SalMotifDB (89). The database also includes information from literature related to the participation of genes and/or gene products in the immune response of teleost fish. This information describes the impact of the infection on different organs of the fish, such as liver, HK, skeletal muscle, and spleen, as the upregulation of genes related to cellular, humoral, innate, and adaptive immune response, cytoskeleton rearrangement, metabolism, and apoptosis, among others. A detailed description of the generation of SalSaDB is available in (**Supplementary Materials and Methods**). The SalSaDB database is available on GitHub for public use (https://github.com/SebastianReyesCerpa/SalSaDB).

### 2.6 GRN analysis

To understand the regulatory cascades underlying the gene expression response to *P. salmonis* infection, we extracted regulatory interactions and gene attributes from SalSaDB for all DEGs and generated infection context-dependent GRNs. Three different GRNs were generated, including only genes regulated at 24-hpi, genes regulated at 48-hpi, and a core GRN including genes consistently regulated at 24- and 48-hpi. Edges in the GRNs were complemented with co-expression information obtained by WGCNA analysis. We determined node degrees in each GRN to identify central regulators (highest outdegree values) using Gephi v0.10 (88, 90). We included in the GRNs as node attributes literature information on gene function in macrophage polarization, differentiation, and function.

## 3 Results

### 3.1 Comparative analysis of transcriptomic response from SHK-1 cells infected with *P. salmonis* against previous organ-level transcriptomic analysis shows a novel set of genes specific to macrophage response

**Figure 1:**
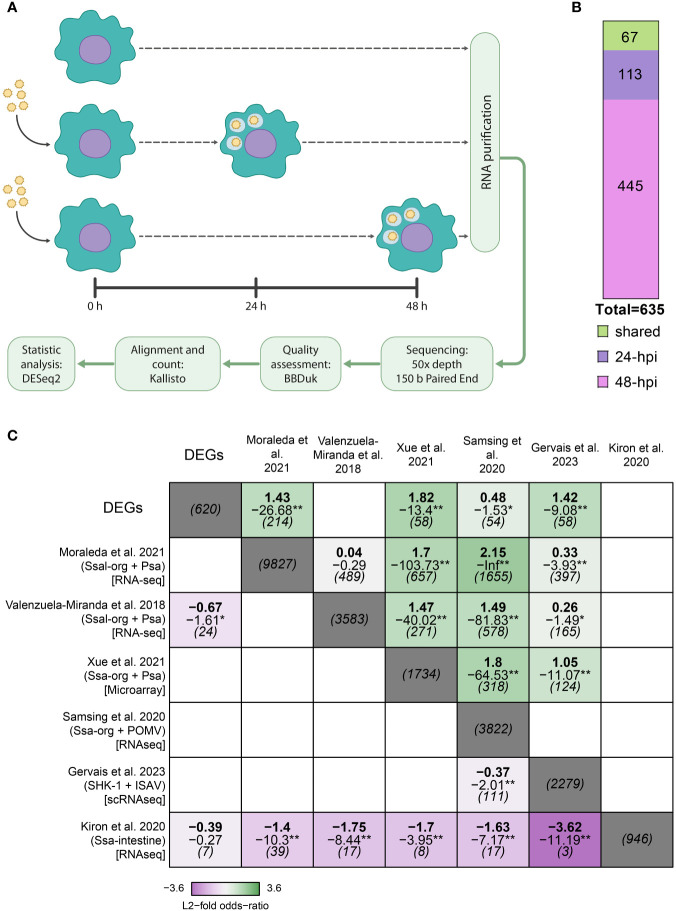
Experimental design and comparative analysis of transcriptomic response in Atlantic salmon challenged with *P. salmonis*. **(A)** Experimental strategy. **(B)** Quantification of DEGs at 24- and 48-hpi, shared and exclusively expressed in each experimental condition. **(C)** Comparison of our DEGs with other studies using GeneSectR (78, 79) where Atlantic salmon immune response was used (Ssa-org: Atlantic salmon tissue analysis; Psa: infection with *P. salmonis*; POMV: infection with *Pilchard orthomyxovirus*; ISAV: infection with *Infectious Salmon Anaemia virus*), obtaining significance of the overlap of genes shared between the different studies. A study about intestine Atlantic salmon without infection feed with different diets was used as an outlier. The upper value is the log2 fisher odds ratio, the middle value is the log10 p-adj, and the lower is the overlap size between datasets. *padj < 0.05, **padj < 0.01. Results >1 log2 transformed Fischer odds ratio along with a log10 adjusted p-value of -1.3 indicates an adjusted p-value of 0.05.

To understand the regulatory mechanism underlying the response of *S. salar* macrophages to *P. salmonis* infection, we infected the macrophage-like cell line SHK-1 for 24- and 48-hpi. We chose these time points because they represent the early stages of infection, before the appearance of characteristic cytopathic effects (CPE), which have been reported since 72-hpi (19, 71). Transcriptomics analysis was performed by RNA-Seq, and differential gene expression was determined using DESeq2 (Materials and Methods) (**Figure 1A**). We obtained 635 differentially expressed genes (DEGs), of which 113 belong exclusively to 24-hpi, 445 to 48-hpi, and 67 were shared between both conditions (**Figure 1B** and **Supplementary File 3**).

Most analyses of the Atlantic salmon response against *P. salmonis* have been performed at the tissue level, probably masking critical processes occurring specifically inside macrophages. To address the relationship between our transcriptomic profile and the transcriptomic profile obtained in other studies, we compared our list of DEGs obtained after RNA sequencing with the results from six previous articles. These include three datasets from Atlantic salmon infected with *P. salmonis* in which the authors analyzed the response in the head kidney, liver, and spleen (27, 50, 80). To gain insights into how specific the response was to *P. salmonis*, we also compared the response of Atlantic salmon to two common viral pathogens, *Infectious salmon anemia virus* (ISAv) and another from SHK-1 cells infected with *Pilchard orthomyxovirus* (POMV) (81, 82) (**Figure 1C**, **Supplementary Table 1**). We also included a non−infection dataset as an unrelated control, consisting of DEGs obtained from Atlantic salmon intestine feed with different diets (83).

To determine how similar our list of DEGs was compared to these datasets, we used a one-tailed Fisher Exact test implemented in the GeneSectR tool. This tool calculates a log2 transformed Fisher-odds ratio between two gene lists, indicating how strong the association between two datasets is, and a log10 adjusted p-value, indicating the significance of that association. Our results suggest that the DEGs observed are similar to those obtained in four of five studies analyzed, regardless of whether they were obtained from Atlantic salmon tissue or SHK-1 cell line or if the infection was carried out with *P. salmonis* or with virus (**Figure 1C**) (80–82, 91). Altogether, the studies that were similar to our DEGs found KEGG pathways or GO terms enrichment related to inflammatory response, programmed cellular death, phagocytosis, and metabolism, and some of the datasets also show RNA metabolism, extracellular matrix and cytoskeletal organization (80–82, 91).

Intriguingly, we found a less-than-expected overlap between our DEGs and those obtained by Valenzuela-Miranda et al. from the spleen and head kidney of Atlantic salmon infected by *P. salmonis*, which may be related to the specific set of genes that were found in this study, which is highly related to amino acids metabolism and little representation of other processes as immune response and cellular differentiation (50). Finally, all the overlaps with the unrelated list of DEGs (83) were less than expected by chance (**Figure 1C**).

Interestingly, we were able to obtain 320 DEGs that were not previously found in the literature (**Supplementary Figure 1**). We performed a functional GO term enrichment analysis on those genes, finding that the associated functions are related to myeloid cell differentiation (GO:0030099) and serine-type carboxypeptidase activity (GO:0004185). In summary, the transcriptomic analysis carried out in our research shows a common set of genes with previous analysis performed in the tissue of infected fish and also uncovers novel DEGs that are exclusively found in SHK-1 macrophage-like cell line infected by *P. salmonis* and that are related to myeloid cell differentiation, a characteristic process of macrophages during its maturation stages to confront a stimulus, as in this case is the infection.

### 3.2 Global differential gene expression analysis in SHK-1 macrophage-like cell line infected by *P. salmonis*

**Figure 2:**
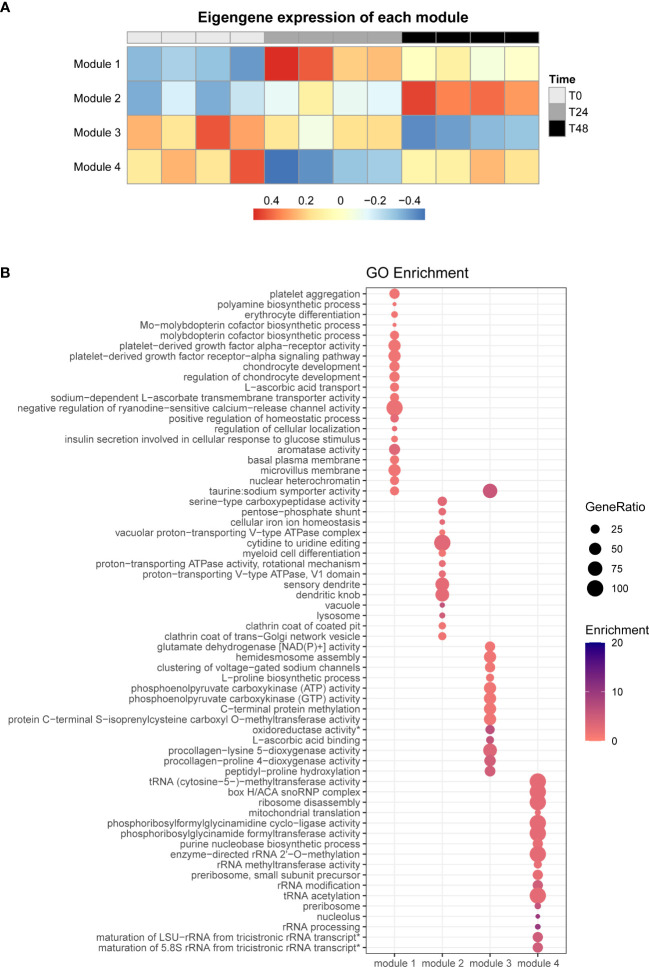
Co-expression analysis of all DEGs and GO terms enrichment analysis. **(A)** WGCNA analysis of all obtained DEGs, rows represent the different co-expression modules, and columns indicate the different infection times analyzed. **(B)** GO term enrichment analysis for each co-expression module, the size of each point is proportional to the gene ratio calculated between the obtained genes from a determinate GO term and the total genes that have that GO term in its annotation; Enrichment represents the -log10(adjusted p-value).

To determine gene expression patterns of a group of genes in the SHK-1 macrophage-like cell line infected by *P. salmonis*, we performed a co-expression analysis using the R package WGCNA (85). We obtained four different modules of genes that shared a similar expression pattern (**Figure 2A**), showing genes that are upregulated at 24- and 48-hpi (module 1), genes that are predominantly upregulated at 48-hpi (module 2), genes that are downregulated mainly at 48-hpi (module 3), and genes that are only downregulated at 24-hpi (module 4) (**Figure 2A**).

To identify the function of the DEGs of each module, we performed a gene ontology (GO) pathway enrichment analysis using the ClueGO platform in Cytoscape (**Figure 2B** and **Supplementary File 4**). We observed that the genes present in module 1 mainly were involved in metabolism (GO:0006596 [ornithine decarboxylase 1 (ODC1)], GO:0006777 [gephyrin A (GPHNA)]), cell differentiation (GO:0030218 [solute carrier family 25 member 38b(SLC2538B)]), and transmembrane transportation (GO:0015882 [solute carrier family 23 member 2-like (SLC23A2)], GO:0070890 [SLC23A2], GO:0005369 [solute carrier family 6 member 6 (SLC6A6)]), platelet aggregation (GO:0005018 [PDGFRA]), regulation of chondrocyte development (GO:0061181 [RFLNA]), aromatase activity (GO:0070330 [CYP1B1, CYP1A]), and insulin secretion involved in cellular response to glucose (GO:0035773 [ANO1]). On the other side, the most significant biological term associated with DEGs in module 2 were associated with processes related to endocytic pathway, such as endocytosis (GO:0030130 [clathrin heavy chain 1 (CLTCL1), clathrin light chain A (CLTA)], GO:0030132 [CLTCL1, CLTA]), proton transportation (GO:0016471 [ATPase H+ transporting V1 subunit G1 (ATP6V1G1), V-type proton ATPase subunit H (VMA13)], GO:0033180 [V-type proton ATPase subunit S1-like (VAS1), V-type proton ATPase catalytic subunit A (ATP6V1A), VMA13], GO:0046961 [VAS1, ATP6V1A, VMA13]), and lysosome (GO:0005764 [cathepsin D (CTSD), prosaposin (PSAP), lysosome-associated membrane glycoprotein 1-like (LAMP1), cathepsin B (CTSB)]). Similarly, in module 3, the GO terms are associated with metabolic process (GO:0016706 [prolyl 4-hydroxylase subunit alpha-1 (P4HA1), procollagen-lysine,2-oxoglutarate 5-dioxygenase 1a (PLOD1) procollagen-lysine,2-oxoglutarate 5-dioxygenase 2-like (PLOD2), prolyl 4-hydroxylase subunit alpha-2 (P4HA2)], GO:0004353 [glutamate dehydrogenase, mitochondrial (GDH)], GO:0004671 [protein-S-isoprenylcysteine O-methyltransferase-like (ICMT)]), and processes related to collagen formation (GO:0004656 [P4HA1B, P4HA2, P4HA1], GO:0008475 [PLOD2, PLOD1]). Finally, module 4 is involved with RNA metabolism (GO:0000463 [BOP1 ribosomal biogenesis factor (BOP1)], GO:0000154 [FtsJ RNA 2’-O-methyltransferase 3 (FTSJ3), N-acetyltransferase 10 (NAT10)], GO:0016428 [RNA cytosine-C(5)-methyltransferase NSUN2-like (NSUN2)]) (**Figure 2B** and **Supplementary File 4**).

In summary, we found two different sets of genes that are differentially expressed in the early stages of macrophages infected by *P. salmonis*, as genes differentially expressed mainly at 24-hpi (modules 1 and 4) and genes differentially expressed predominantly at 48-hpi (modules 2 and 3). The set of genes differentially expressed mainly at 24-hpi are related to an upregulation in genes related to metabolism, cell differentiation, cell projection, and transmembrane transportation, processes in which the participant genes mainly belong to the solute carrier families; at 24-hpi, we also observed a downregulation in genes related to RNA metabolism. On the other hand, at 48-hpi, we observed an upregulation of genes classified in module 2 related to endocytosis and lysosome, characterized by clathrin-associated genes and several V-type ATPases and cathepsin genes, which are involved in different steps of the endocytic pathway, from the internalization of the extracellular material to the acidification of the vacuole by the action of the V-ATPases; as for the module 3, the downregulated genes are related to metabolism, characterized by some genes related to carbon and collagen metabolism, associated to the extracellular matrix metabolism.

### 3.3 Gene regulatory network analysis: SalSaDB

Our results indicate that *P. salmonis* infection of macrophages elicits significant transcriptomic reprogramming. We next sought to identify the regulatory mechanisms underlying these changes by constructing a context-specific GRN model of TF-target gene interaction between our DEGs. In order to construct this context-specific GRN, we first assembled a reference GRN including all available information on TFs and target genes for Atlantic salmon obtained from SalMotifDB (89). This network represents all the possible regulatory interactions between TFs and target genes, irrespective of developmental stage, tissue, or experimental condition, so we call it a “reference” network (92). This reference GRN is available in the GitHub of SalSaDB (https://github.com/SebastianReyesCerpa/SalSaDB).

SalSaDB contains gene name equivalence from NCBI to ENSEMBL for ~76% of the genes from Atlantic salmon and automatically obtained gene symbol information for ~24% of the genes. We manually curated annotations for our list of DEGs, achieving ~99% of gene information data for our DEGs (**Table 1**). The reference GRN contains ~66% of the annotated genes in version 3.1 of the genome of Atlantic salmon, with 39,048 nodes corresponding to genes and 10.8 million edges corresponding to regulatory interactions between TFs and target genes.

**Table 1:** SalSaDB statistics.

Origin database	Number of NCBI_GID assigned	Percentage
ENSEMBL (automatic)	32.509	75,63%
ENSEMBL (manual)*	45	69.23%
Gene Symbol - code	32.632	75,91%
Gene Symbol - abbreviation	10.354	24,09%
Gene Symbol – abbreviation (manual)*	435	98.46%
Product	42.986	100,00%
Product - uncharacterized	2.334	5,43%
Transcript	95.307	221,72%
Microarray PID	2.380	5,54%

*The values for manual curation only consider differentially expressed genes (at 24 and/or 48 hpi).

### 3.4 KLF17 controls the GRNs of SHK-1 macrophage-like cells infected by *P. salmonis*

To identify the regulatory cascades that control gene expression response of the SHK-1 cell line infected by *P. salmonis* at 24- and 48-hpi, which could lead to an ineffective macrophage response explaining bacterial survival, we used the information in our reference GRN to generate context-specific GRNs containing our DEGs as nodes. The GRN obtained using the 24-hpi DEGs is composed of 175 nodes and 321 edges, with the participation of 15 TFs, from which KLF17 possesses the highest outdegree connectivity (73 regulated nodes) (**Table 2** and **Supplementary File 5**). As for the GRN generated using the 48-hpi DEGs, it is composed of 486 nodes and 1286 edges, regulated by 20 TFs, from which, again, KLF17 is the TF with the highest outdegree connectivity (189 regulated nodes) (**Table 2** and **Supplementary File 5**). To identify the maintained regulatory interactions between TFs and target genes at 24- and 48-hpi, we analyzed the shared response between 24- and 48-hpi; we obtained a core GRN, which is integrated by 65 nodes and 85 edges, with 7 TFs in total, highlighting KLF17 as the TF with the highest outdegree connectivity (31 regulated nodes) (**Table 2** and **Supplementary File 5**).

**Table 2:** GRNs statistics.

Attribute	Reference	24-hpi	48-hpi	Core
**Nodes**	39,048	175	486	65
**Edges**	10,763,461	321	1286	85
**TFs**	1,848	15	20	7
**Max outdegree**	15450 (bcl11aa)	73 (KLF17)	189 (KLF17)	31 (KLF17)
**Genes in the GRN (all)**	65.66%	0.29%	0.82%	0.11%

To classify the TFs in groups related to the M1− or M2−like phenotype of macrophages, we manually assigned the relation of each TF in our DEGs with one phenotype according to bibliography (human, mouse, and zebrafish) (**Table 3**), and based on the expression pattern, we assigned an expected effect in the macrophage polarization according to our results (e.g., if a TF is related to M1−like phenotype and is downregulated, then the expected effect is to promote M2−like phenotype; **Table 3**). As for the TFs found in the core GRN, we found that KLF17 (also annotated as KLF4), MAFBA, WT1B, AHR2B, and DLX3B were related to an M2−like phenotype and were upregulated, suggesting an effect towards M2−like phenotype (93–98). On the other hand, NOTCH3 and NFATC1 were related to the M1−like phenotype, but as those genes were downregulated, we expect an effect toward the M2−like phenotype (99, 100). At 24−hpi, we found that ZFHX3, LEF1, and TBX2B were associated with an M2−like phenotype, which matched the expected effect over macrophage polarization, in the same way as the TFs related to M1−like phenotype, such as ARID3A, IRF1, and BNC1 (101–108). Finally, the TFs found at 48−hpi as CBFB, CREB1B, CREB3L2, and GLIS1B were related to an M2-like phenotype (109–113), but as those TFs were downregulated, their expected relation was assigned toward M1−like phenotype. HIVEP2A, associated with an M1−like phenotype, was assigned to an M2−like phenotype (114) as it is downregulated. Finally, the TFs associated with an M2−like phenotype that were upregulated, thus conserving its role, were SREBF1 (two copies), ZNF410, and SREBF2 (115–117).

**Table 3:** Classification of TFs in macrophage polarization.

TF	Product	Expression	Polarization	Expected effect in polarization	Condition	Reference	DOI	Model
**KLF17**	Krueppel-like factor 4	UP	M2	M2	Core	2011 - Liao	10.1172/JCI45444	mice
**WT1B**	WT1 transcription factor b	UP	M2	M2	Core	2019 - Sanz-Morejón	10.1016/j.celrep.2019.06.091	zebrafish
**NOTCH3**	neurogenic locus notch homolog protein 2-like	DOWN	M1	M2	Core	2020 - López-López	10.1038/s41598-020-71810-4	mice
**NFATC1**	Nuclear factor of activated T cells 1	DOWN	M1	M2	Core	2017 - Klein-Hessling	10.1038/s41467-017-00612-6	mice
**MAFBA**	v-maf avian musculoaponeurotic fibrosarcoma oncogene homolog Ba	UP	M2	M2	Core	2020 - Hamada	10.1538/expanim.19-0076	mammals (mice, human, mouse)
**FOXF1**	forkhead box protein F1	UP	UNKNOWN	UNK	Core			
**AHR2B**	aryl hydrocarbon receptor 2 beta	UP	M2	M2	Core	2018 - Climaco-Arvizu; 2020 - Yang	10.1016/j.lfs.2016.05.001 ; 10.7150/thno.51144	mice
**DLX3B**	distal-less homeobox gene 3b	UP	M2	M2	Core	2011 - Hwang	10.1073/pnas.1019658108	mouse
**ZFHX3**	zinc finger homeobox protein 3	UP	M2	M2	24-hpi	2023 - Casalino-Matsuda (preprint)	10.1101/2023.02.28.530480	mice
**ARID3A**	AT rich interactive domain 3A (BRIGHT-like)	UP	M1	M1	24-hpi	2019 - Ratliff	10.1016/j.jaut.2018.09.013	human
**LEF1**	lymphoid enhancer-binding factor 1	UP	M2	M2	24-hpi	2021 - Luquero	10.1111/jcmm.16723	human
**IRF1**	interferon regulatory factor 1	UP	M1	M1	24-hpi	2016 - Xie	10.3892/ijmm.2016.2583	human
**BNC1**	zinc finger protein basonuclin-1; Basonuclin 1	UP	M1	M1	24-hpi	2022 - Liang	10.1177/15353702211052036	human
**TBX2B**	T-box transcription factor 2b	UP	M2	M2	24-hpi	2023 - Truxova	10.1136/jitc-2022-005968	human
**MXD4**	MAX dimerization protein 4	UP	M1	M1	24-hpi	2017 - Lin; 1993 - Ayer	10.18632/oncotarget.21510 ; 10.1101/gad.7.11.2110	mouse; human
**CBFB**	core-binding factor subunit beta	DOWN	M2	M1	48-hpi	2019 - Malik; 2019 - Nowak	10.1038/s41467-019-10102-6 ; 10.1016/j.ebiom.2019.10.063	human (by regulation of EIF4B)
**SREBF1**	Sterol regulatory element binding transcription factor 1	UP	M2	M2	48-hpi	2021 - Bidault	10.1038/s42255-021-00440-5	mouse
**CREB1B**	cAMP responsive element binding protein 1b	DOWN	M2	M1	48-hpi	2021 - Polumuri	10.1177/1753425920975082	mice
**ZNF410**	Zinc finger protein 410	UP	M2	M2	48-hpi	2002 - Benanti	10.1128/MCB.22.21.7385-7397.2002	human
**TBX3A**	T-box transcription factor 3a	DOWN	UNKNOWN	UNK	48-hpi			
**GLIS1B**	GLIS family zinc finger 1b	DOWN	M2	M1	48-hpi	2021 - Chen	10.3389/fimmu.2021.688910	mammals (mice, mouse)
**SREBF2**	sterol regulatory element binding transcription factor 2	UP	M2	M2	48-hpi	2019 - Kusnadi	10.1016/j.immuni.2019.06.005	mice
**NR2E1**	nuclear receptor subfamily 2 group E member 1	UP	UNKNOWN	UNK	48-hpi			
**CREB3L2**	cyclic AMP-responsive element-binding protein 3-like protein 2	DOWN	M2	M1	48-hpi	2015 - Luan	10.1073/pnas.1519644112	human
**TEFA**	thyrotroph embryonic factor	DOWN	UNKNOWN	UNK	48-hpi			
**HIVEP2A**	HIVEP zinc finger 2a	DOWN	M1	M2	48-hpi	2016 - Lu	10.1038/srep37446	mice

### 3.5 Functional analysis of differentially expressed target genes regulated by the TFs reveal biological process associated with metabolism, RNA metabolism, cytoskeletal remodeling, endocytosis, and lysosomal response

**Figure 3:**
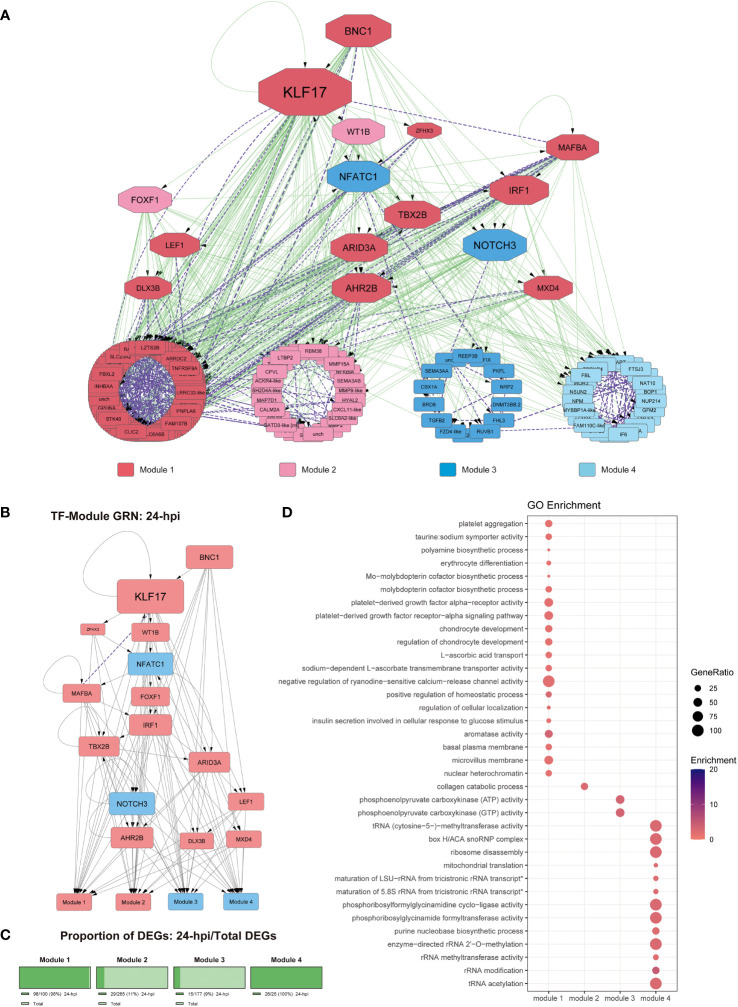
Gene regulatory network at 24-hpi. **(A)** Co-expression network along with regulatory interactions obtained from our reference GRN. Each module is color-coded, module 1: genes upregulated since 24-hpi; module 2: genes upregulated at 48-hpi; module 3: genes downregulated at 48-hpi; module 4: genes downregulated at 24-hpi. Purple, undirected purple dashed edges represent co-expression and directed green edges represent TF-target regulatory interactions. The transcription factors are represented as octagons. **(B)** GRN of all the DEGs at 24-hpi, the nodes are color-coded to match upregulation (red) and downregulation (blue). The target genes are represented as modules for ease of view of TF hierarchy and regulation. Purple, undirected purple dashed edges represent co-expression, while directed grey edges represent TF-target regulatory interactions. **(C)** Abundance of DEGs in percentage between 48-hpi DEGs and the DEGs found in our global analysis. **(D)** Functional analysis by GO term enrichment performed for each co-expression module (horizontal axis). The size of each point is proportional to the gene ratio calculated between the obtained genes from a determinate GO term and the total genes that have that GO term in its annotation; Enrichment represents the -log10(adjusted p-value).

**Figure 4:**
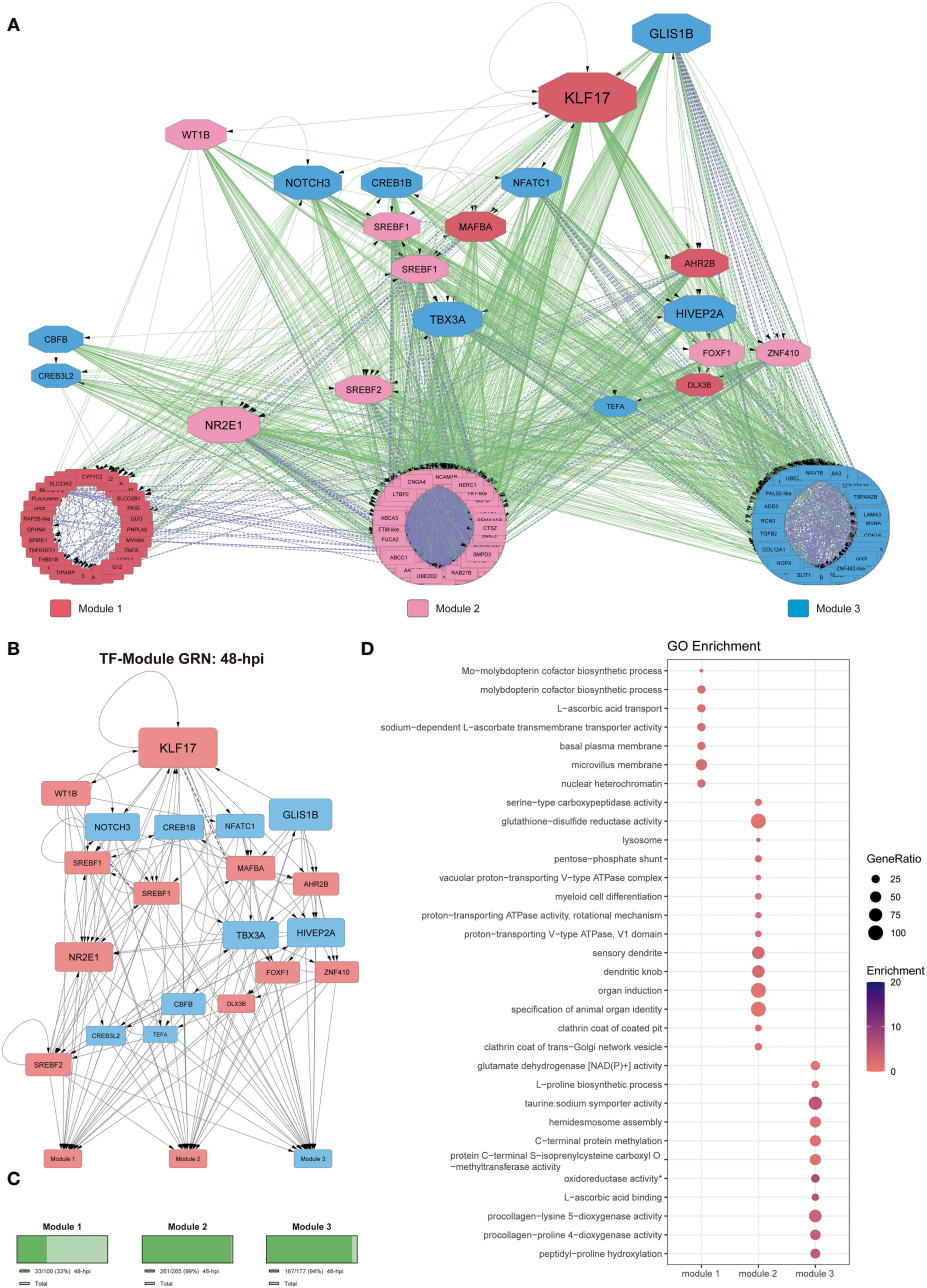
Gene regulatory network at 48-hpi. **(A)** Co-expression network along with regulatory interactions obtained from our reference GRN. Each module is color-coded, module 1: genes upregulated since 24-hpi; module 2: genes upregulated at 48-hpi; module 3: genes downregulated at 48-hpi. Purple, undirected purple dashed edges represent co-expression and directed green edges represent TF-target regulatory interactions. The transcription factors are represented as octagons. **(B)** GRN of all the DEGs at 48-hpi, the nodes are color-coded to match upregulation (red) and downregulation (blue). The target genes are represented as modules for ease of view of TF hierarchy and regulation. Purple, undirected purple dashed edges represent co-expression, while directed grey edges represent TF-target regulatory interactions. **(C)** Abundance of DEGs in percentage between 48-hpi DEGs and all the DEGs found in our global analysis. **(D)** Functional analysis by GO term enrichment performed for each co-expression module (horizontal axis). The size of each point is proportional to the gene ratio calculated between the obtained genes from a determinate GO term and the total genes that have that GO term in its annotation; Enrichment represents the -log10(adjusted p-value).

We performed a GO term enrichment analysis to examine the functional context of the target genes regulated by the TFs observed in our GRN. Then, to obtain a better context of those results, we classified the results by the upstream hierarchy of the obtained GO terms. Moreover, to provide a better context for the regulatory cascade from the different GRNs, we hierarchically organized the TFs, kept the classification from the previous co-expression analysis of the DEGs (**Figure 2**), and grouped the regulated genes by those modules. We also quantified the percentage of genes present in each module from the DEGs observed at 24- and 48-hpi to determine how represented each module is at both infection times analyzed (**Figures 3A**, **4A**).

The connectivity analysis of this GRN shows that KLF17 is an important regulatory component of this context-specific GRN (**Figures 3A, B**). As for the functional analysis of the DEGs from each module at 24-hpi, we observed that DEGs involved with module 1, which were upregulated and co-expressed with most upregulated TFs, represent ~98 of this module (**Figure 3C**). These DEGs are involved with functions related to cell differentiation (GO:0030218), signal transduction (GO:0070527, GO:0035790, GO:0005018), basal plasma membrane (GO:0009925), microvillus membrane (GO:0031528), and transmembrane transportation (GO:0005369, GO:0015882) (**Figure 3D**); module 2 (upregulated, ~11%; **Figure 3C**) was related to collagen catabolic process (GO:0030574) (**Figure 3D**); module 3 (downregulated, ~9%; **Figure 3C**) was co-expressed with downregulated TFs and showed processes related to carbon metabolism (GO:0004612, GO:0004613); module 4, which is downregulated and it is an exclusive module for 24-hpi, were related mainly to RNA metabolism (GO:0051391, GO:0000154, GO:0008649, GO:0000453, GO:0032790, GO:0000463, GO:0000466) and carbon metabolism (GO:0004641, GO:0004644) (**Figure 3** and **Supplementary File 6**).

At 48-hpi, the connectivity analysis shows that KLF17 is also an important regulator (**Figures 4A, B**). As for the functional analysis of the DEGs from each module at 48-hpi, we observed that the genes involved in module 1 (upregulated, ~33%; **Figure 4C**) were mainly related to metabolism (GO:0006777, GO:0032324, GO:0015882), basal plasma membrane (GO:0009925), microvillus membrane (GO:0031528), and transmembrane transportation (GO:0015882, GO:0070890). The DEGs grouped in module 2 (upregulated, ~99%; **Figure 4C**) were related to cell differentiation (GO:0030099, GO:0010092), proton transportation (GO:0033180, GO:0016471), lysosome and trans-Golgi network (GO:0005764, GO:0030130), endocytosis (GO:0030132, GO:0030130), and transmembrane transportation (GO:0046961). DEGs from module 3 (upregulated, ~94%; **Figure 4C**) are related to transmembrane transportation (GO:0005369), metabolism in general (GO:0006481, GO:0019511, GO:0004353, GO:0004671, GO:0016706), and collagen metabolism (GO:0008475, GO:0004656) (**Figure 4D** and **Supplementary File 7**).

**Figure 5:**
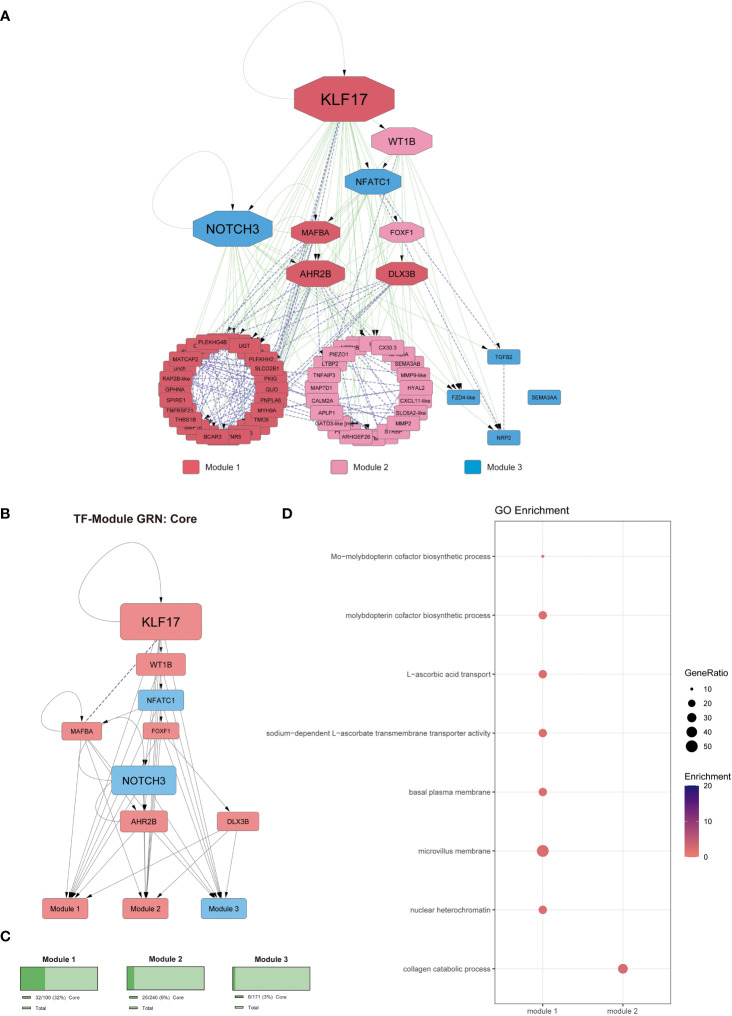
Gene regulatory network for core genes. **(A)** Co-expression network along with regulatory interactions obtained from our reference GRN. Each module is color-coded, module 1: genes upregulated since 24-hpi; module 2: genes upregulated at 48-hpi. Purple, undirected purple dashed edges represent co-expression and directed green edges represent TF-target regulatory interactions. The transcription factors are represented as octagons. **(B)** GRN of all the core DEGs, the nodes are color-coded to match upregulation (red) and downregulation (blue). The target genes are represented as modules for ease of view of TF hierarchy and regulation. Purple, undirected purple dashed edges represent co-expression, while directed grey edges represent TF-target regulatory interactions. **(C)** Abundance of DEGs in percentage between core DEGs and all the DEGs found in our global analysis. **(D)** Functional analysis by GO term enrichment performed for each co-expression module (horizontal axis). The size of each point is proportional to the gene ratio calculated between the obtained genes from a determinate GO term and the total genes that have that GO term in its annotation; Enrichment represents the -log10(adjusted p-value).

Finally, our core GRN was generated by selecting the DEGs shared between 24- and 48-hpi, we delimited our search of relevant genes to less than 100, which allowed us to put more effort into contextualizing the role of those genes in the macrophage response while also getting the TFs that control the regulatory cascade through both times analyzed. This information allowed us to determine the process involved in the establishment of infection of *P. salmonis* in SHK-1 cells that even show the same expression pattern, up- or down-regulated at both times analyzed, showing a consistent response through early infection times. In this GRN, we also observed that KLF17 is an important regulator with the highest outdegree connectivity (**Figures 5A, B**). As for the functional analysis of each module from core DEGs (overlap between 24- and 48-hpi), we observed that genes from module 1 (upregulated, ~32%) were related to metabolism (GO:0006777), plasma membrane projection (GO:0031528, GO:0009925), and transmembrane transportation (GO:0015882, GO:0070890). The genes in module 2 (upregulated, ~9%) were related to the collagen catabolic process (GO:0030574), and genes co-expressed in module 3 did not show significant results from the functional analysis (**Figure 5** and **Supplementary File 8**).

**Figure 6:**
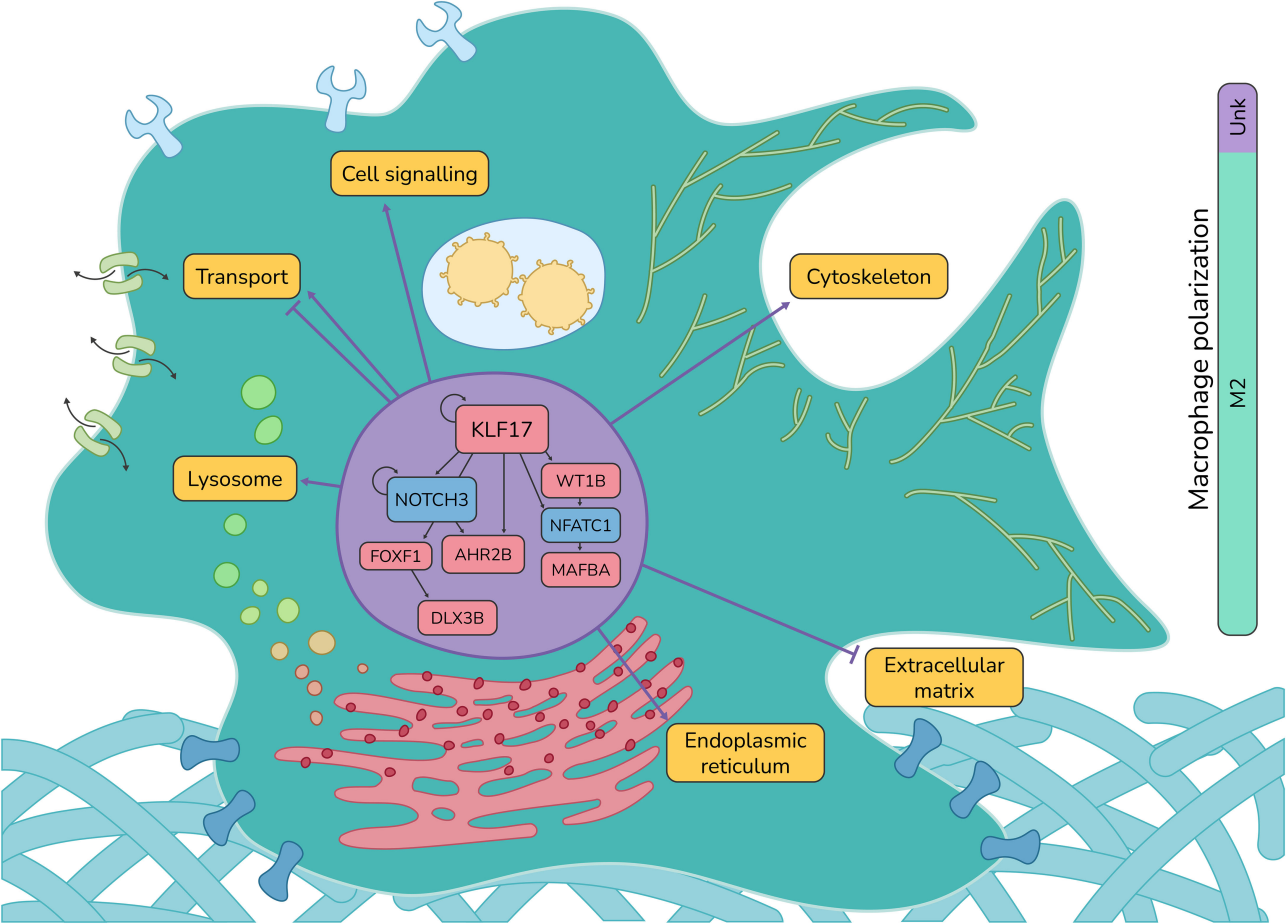
Atlantic salmon macrophage response against *P. salmonis*. Summary of the regulatory interactions between TFs and the biological processes they regulate in Atlantic salmon macrophage-like cells SHK-1 infected by *P. salmonis*.

Altogether, our results show that the SHK-1 macrophage-like cell line infected by *P. salmonis* shows a regulatory cascade with KLF17 as an essential regulator at early times of infection. This cascade is also integrated by NOCH3, FOXF1, DLX3B, AHR2B, WT1B, NFATC1, and MAFBA, whose we could classify as M1-like related TFs and M2-related TFs. The M1-related TFs are NOTCH3 and NFATC1, which are downregulated, thus not promoting an M1-like phenotype. The TFs related to an M2-like phenotype are KLF17, DLX3B, AHR2B, WT1B, and MAFBA, which are upregulated and promote differentiation toward an M2-like phenotype (**Figure 6**).

## 4 Discussion

The understanding of the specific transcriptomic response of Atlantic salmon macrophages against *P. salmonis* has yet to be well explored because most analyses have been performed at the tissue level, probably masking critical processes occurring specifically inside macrophages. First, we determined how much we contributed to the state of the art of Atlantic salmon macrophage immune response with our transcriptomic analyses in macrophage-like cells (SHK-1) infected with *P. salmonis*. Although we found 320 DEGs that were not previously found in the literature and are related to the myeloid cell differentiation process, which has been observed in macrophages stimulated by pathogen-associated molecular patterns (PAMPs) from *P. salmonis* (118, 119) (**Supplementary Figure 1**), the biological process in which they are involved were also found in other studies of Atlantic salmon infected by *P. salmonis*, as is downstream the terms of cell differentiation and cell development (21, 22, 91).

Interestingly, in Gervais et al. (81), the authors analyze the transcriptomic response of SHK-1 challenged by ISAV by RNA-seq, obtaining 2279 DEGs, of which only 58 are shared genes with our results. However, in Moraleda et al. (80), the authors also analyze by RNA-seq the transcriptomic response of the head kidney and spleen from Atlantic salmon infected by *P. salmonis*, obtaining 9827 DEGs, of which 214 are shared with our DEGs, which corresponds to 34,5% of our DEGs are shared with those reported by Moraleda et al. (80). Conversely, only 9,35% of our DEGs are shared with those reported by Gervais et al. (**Figure 1C**), suggesting that our study seems to show a more shared response with the studies in which the transcriptomic response was analyzed by RNA-seq in Atlantic salmon challenged with *P. salmonis*, rather than a response against viruses infecting the same macrophage-like cell line (81). Moreover, the DEGs shared between our research and the previously reported DEGs are statistically similar, suggesting that in our results, we also found genes commonly expressed in Atlantic salmon tissues when infected by *P. salmonis*. Altogether, the processes observed in the other studies performed in Atlantic salmon infected by *P. salmonis* have shown that the DEGs were related to amino acid metabolism, adaptive immune response, phagocytosis, apoptosis, cytoskeleton, and intracellular trafficking, biological processes that were also observed in our results (**Figures 3**–**5** and **Supplementary Files 6**-**8**), supporting this significant overlap between the different datasets (50, 80, 91).

The co-expression analysis of our DEGs allowed us to categorize our genes into four modules, which helped us to analyze the functionality of those genes by groups; in this way, we achieved a detailed assignation of functions by modules (**Figure 2**), gaining depth in our analysis, as it can be compared with our previous result, when we analyzed our unique 320 DEGs, in which we could not obtain detailed information performing the same analysis. As we observed the expression patterns of our DEGs in the WGCNA analysis, we noticed that some modules were almost exclusive for each condition, such as modules 2 and 3 at 48−hpi and modules 1 and 4 at 24−hpi; that was confirmed when we calculated the percentage of genes present in each module for each time of infection analyzed (**Figures 2**–**4**). We also observed the conservation of the assigned functions by the GO term enrichment analysis between all the genes present in each module and the further analysis for the gene modules specific for 24− and 48−hpi (**Figures 2**–**4** and **Supplementary Files 3**-**5**). Interestingly, we found several processes that are related to M2 phenotype polarization, such as platelet aggregation, reportedly related as a product of M2 differentiation (120); development of chondrocytes, a process associated with collagen secretion, that also is associated with M2 macrophage differentiation (121); and insulin secretion response to glucose which has been linked to M2 activation by an increase of sensitivity to glucose (122). Nevertheless, we also observed several processes linked to M1 macrophage polarization, such as aromatase activity, a process linked to an inflammatory response by the induction of aromatase in pair with the development of an inflammatory response (123); procollagen activity, a process involved in the synthesis of collagen, and as the genes related to this are downregulated, we might assume a decrease in the collagen synthesis (124). Altogether, these results suggest that macrophages infected by *P. salmonis* after 24-hpi present a phenotype towards M2-like macrophage spectrum by the proportion of processes associated with each phenotype.

In the functional analysis of the DEGs at 24-hpi, we found that the genes belonging to module 1 were related to processes, such as cell differentiation, membrane process, and transmembrane transportation, while the genes grouped in module 2 were mainly related to collagen metabolism and genes co-expressed in module 3 were related to carbon metabolism. All the biological processes grouped in both module 1 and module 2 may be associated with the induction of endocytosis of *P. salmonis* through the modulation of the clathrin and actin cytoskeleton (**Figure 3**) (13, 125, 126). On the other hand, the genes grouped in module 4 (downregulated) were related mainly to RNA metabolism, suggesting that the bacteria promotes RNA metabolism (**Figure 3**), which could be a signal of post-transcriptional gene regulation or even transcriptional regulation mediated by miRNAs, mechanisms previously reported in mammals hosts infected by other intracellular pathogens, as mice infected by *Pseudomonas aeruginosa*, and human HeLa cells infected by *Salmonella enterica.* Moreover, this has also been suggested in Atlantic salmon infected by *P. salmonis*, raising the possibility of further investigating the non-coding RNAs in the macrophage immune response of Atlantic salmon (51, 127, 128).

In macrophages, after 48-hpi, we found several enriched processes that reinforce the idea of the imbalanced membrane transportation in infected macrophages, such as podosome, cell projection membrane, clathrin-related processes, and lamellipodium (**Figure 4**), along with endocytic/phagocytic vesicle transportation, as a lysosome-related process, vacuole, small GTPases (such as Rab proteins), and v-ATPases (characteristic of lysosomal acidification) (**Figure 4**). Those results correlate with previous findings, where *P. salmonis* was found to imbalance the endocytic pathway of infected macrophages, partially inactivating the lysosomal acidification and its degradation capacity (15, 17).

To contribute to the Atlantic salmon transcriptomics community, we created SalSaDB as a new resource to analyze the Atlantic salmon gene expression results. This contribution was one of our most significant achievements due to the lack of easy-to-access information that crosslinks different databases. This work would be trivial using R studio packages that allow users to access this information quickly. However, those resources are currently available for model organisms, such as mice, zebrafish, and cows, but not Atlantic salmon. SalSaDB proved its value in analyzing our data, helping us translate our results obtained using ENSEMBL genomic information to the NCBI genomic information and take advantage of the GRN created using the publicly available information from SalMotifDB (89). Generating the reference GRN for Atlantic salmon resulted in a non-condition-biased result that will be useful to several researchers who need to perform a regulatory analysis in a different context of the Atlantic salmon research field. We quickly observed which genes were relevant in our experimental conditions using the GRN and our dictionary for the gene names and gene product, making efficient analysis of the DEGs found in SHK-1 cells infected by *P. salmonis*. As SalSaDB has the potential to become a helpful resource for the scientific community that studies Atlantic salmon, and with the spirit of making it accessible to researchers, we make it publicly available in GitHub, including a detailed description in the code used to obtain the information and how to use the GRN.

Our transcriptional analysis observed several TFs controlling the gene expression in 24− and 48−hpi and exclusively to each time point analyzed (**Table 3**). First, we found essential TFs in our core GRN, which previously were reported as regulators of macrophage polarization, such as KLF17 and MAFBA (both upregulated) and NFATC1 and NOTCH3 (both downregulated) (**Figure 5**). In mammals (mice and humans), KLF-17 and MAFBA have been associated with an M2-like macrophage profile. Conversely, NFATC1 and NOTCH3 have been associated with M1-like macrophages (94, 96, 99, 100), suggesting that our results in macrophage-like cells SHK-1 infected by *P. salmonis* possibly develop a regulation towards M2-like phenotype.

Additionally, we also categorized the other TFs that regulate the core GRN, which were all upregulated, WT1B, FOXF1, AHR2B, and DLX3B, which have been described to be related to M2-like phenotype in zebrafish (WT1B), mice (AHR2B), and mouse (DLX3B). Conversely, FOXF1 is not described in the context of macrophage polarization (93, 95, 97, 98). Altogether, those results suggest that the core regulation of macrophages infected by *P. salmonis* at early times is tightly controlled by TFs related to an M2-like phenotype (**Figure 6**).

As we analyze the TFs that belongs to each time of infection evaluated, we found that at 24-hpi, more than half of TFs are related to an M1 phenotype in humans and mouse, and also were upregulated in our results, corresponding to ARID3A, IRF1, MXD4, and BNC1. On the other hand, we also observed TFs that in mammals have been associated with an M2-like polarization, which corresponds to ZFHX3, LEF1, and TBX2B (101–108). Similarly, the classification of the TFs at 48-hpi shows that TFs with a known role in macrophage polarization are related to M1-like and M2-like macrophages. The TFs related to an M1-like phenotype in mammals were CBFB (indirectly, by regulating EIF4B), CREB1B, GLIS1B, and CREB3L2 (109–113), while the TFs related to an M2-like phenotype in mammals were two copies of SREBF1, SREBF2, ZNF410, and HIVEP2A (114–117). Although at 48-hpi the macrophage polarization phenotype seems to be shifting to a less M2-like phenotype but maintaining a predominant regulatory cascade related to M2-like macrophages.

To understand the root of the imbalance that *P. salmonis* generates in infected macrophages and its impact on macrophage polarization, differentiation, and function, we used our GRN to unravel the regulatory interaction of the DEGs found in our RNA sequencing. In our work, we select the most relevant genes found in our GRN by connectivity and magnitude of the log2(FC). Additionally, we include a bibliographic review of the previously reported relevance in this process. Our GRNs are relatively large, with 321 and 1,286 edges at 24- and 48-hpi, so we constructed a core GRN of the DEGs shared at both times. This core GRN allows us to find the genes maintained as differentially expressed, which present a similar expression pattern. Altogether, the core genes should be key to modulating the macrophage response in *P. salmonis* infection.

The involvement of TFs belonging to the KLF family has been described previously in the polarization of Atlantic salmon macrophages. A study conducted in primary culture of head kidney leukocytes (HKLs) from Atlantic salmon enriched in macrophage−like populations reported two TFs from the Krüppel−like factors family, KLF2 and KLF9, which were downregulated during monocyte to macrophage differentiation (37). Our results showed an upregulation of KLF17, a TF whose upregulation is negatively associated with cell motility in different human cancer cell lines (129–131). Moreover, the Atlantic salmon KLF17 gene is also annotated as KLF4-like, a characteristic TF of M2 polarized macrophages (96). Together, our results suggest that the upregulation of KLF17 in SHK-1 cells infected by *P. salmonis* could be suppressing the cell motility of the infected macrophages at early infection times and probably promoting an M2-like phenotype in infected macrophages.

Among TFs observed upregulated in our experimental conditions, we observed MAFBA. Some studies link the expression of this TF to an increased proteasome activity and activation of the innate immune response in human cancer cell lines (132, 133). Moreover, this TF has been described as an exclusive macrophage TF, expressed in macrophages polarized to an M2−like phenotype (94). Our results show that MAFBA is upregulated in SHK-1 cells infected by *P. salmonis*. In this context, we can suggest that MAFBA regulates polarization towards an M2-like phenotype in macrophages infected by *P. salmonis*, promoting an innate immune response instead of a specific immune response against infection. On the other hand, NOTCH3 is a macrophage-exclusive TF that positively regulates the NF-κB pathway, which is characteristic of M1-like macrophages (100). Our study showed downregulation of NOTCH3 expression, suggesting an impaired response mediated by NF-κB and an M2-like macrophage profile in SHK-1 cells infected by *P. salmonis*.

## 5 Conclusion

Understanding the regulatory mechanisms of macrophage polarization opens the possibility of modulating the response mediated by macrophages. Our work presents a comprehensive description of the transcriptomic response of macrophages infected by *P. salmonis* with an emphasis on the regulatory cascades involved in macrophage polarization and function. Although we observed changes in the expression of genes associated with an M1-like profile in infected SHK-1 cells, our results suggest that this response is probably not robust and early enough to generate an effective response; on the contrary, we uncovered a regulatory cascade that shifts gene expression towards an M2-like phenotype, affecting diverse effectors related to the lysosome, RNA metabolism, cytoskeleton organization, remodeling of extracellular matrix, and cell migration, among others. These concerted changes in gene expression could explain the survival of *P. salmonis* when it infects Atlantic salmon macrophages (**Figure 6**). Moreover, this work highlights the use of a curated database of TF-target gene interactions for Atlantic salmon, SalSaDB, that is freely available for the scientific community.

## Data availability statement

The datasets presented in this study can be found in online repositories. The names of the repository/repositories and accession number(s) can be found below: PRJNA996662 (SRA).

## Ethics statement

Ethical approval was not required for the studies on animals in accordance with the local legislation and institutional requirements because only commercially available established cell lines were used.

## Author contributions

DP-S: Data curation, Formal analysis, Investigation, Methodology, Software, Validation, Writing – original draft, Writing – review & editing. MF: Investigation, Methodology, Writing – review & editing. VI: Investigation, Methodology, Writing – review & editing. BB: Investigation, Methodology, Writing – review & editing. ES: Data curation, Formal analysis, Methodology, Writing – review & editing. JR-P: Formal analysis, Investigation, Methodology, Supervision, Writing – review & editing. EV-V: Formal analysis, Investigation, Methodology, Writing – review & editing. FR-L: Conceptualization, Formal analysis, Investigation, Methodology, Writing – review & editing. DT-A: Formal analysis, Investigation, Methodology, Writing – review & editing. EAV: Conceptualization, Formal analysis, Funding acquisition, Investigation, Methodology, Supervision, Writing – review & editing. SR-C: Conceptualization, Formal analysis, Funding acquisition, Investigation, Methodology, Project administration, Supervision, Writing – original draft, Writing – review & editing.

## Supplementary material

Supplementary Figure 1 Venn diagram of overlap between DEGs from different studies and ours. Venn diagram shows the overlap between our DEGs and the gene lists obtained from online data from studies about Atlantic salmon infected by *P. salmonis* and the virus. We grouped the DEGs by Ssa_Psa for the gene list from papers about Atlantic salmon infected by *P. salmonis* and Ssa_virus for the gene lists from papers about Atlantic salmon infected by virus (ISAv and POMV).

SUPPLEMENTARY FIGURE 2 Manual gene homologation from NCBI to ENSEMBL databases. Gene ID homologation was carried out by comparison of genomic coordinates in NCBI Genome Data Viewer. The top panel represents a complete overlap between the exons of a gene annotation from both databases. The mid panel shows an example of a partial overlap of exons, in which the observed difference of exons coordinates between both databases and the absence of exons from one database from another. The bottom panel shows the decision flow diagram that was used to assign the homologation of a partial overlap between the gene annotation from NCBI and ENSEMBL, with the priority of databases to assign the gene ID when ENSEMBL external references matched NCBI annotation.

SUPPLEMENTARY FIGURE 3 SalSaDB recompilation diagram. SalSaDB was constructed using information from several databases and research papers to improve Atlantic salmon genomic information for this work. This diagram shows the quantity of genes successfully gathered and linked with NCBI information, with the steps we followed to improve the automatically obtained data for our DEGs.

Supplementary File 1 rRNA sequences of Atlantic salmon.txt.

Supplementary File 2 Adaptors.txt.

Supplementary File 3 Differentially expressed genes (DEGs).

Supplementary File 4 All DEGs GO annotation.

Supplementary File 5 GRN tables from cytoscape.

Supplementary File 6 24-hpi GO annotation.

Supplementary File 7 48-hpi GO annotation.

Supplementary File 8 Core GO annotation.

Supplementary Materials and Methods Description of generation of SalsaDB.
