# Supplementary figures and images for: KLF17 is an important regulatory component of the transcriptomic response of Atlantic salmon macrophages to Piscirickettsia salmonis infection
Source: Front Immunol. 2023 Dec 14;14:1264599. doi: 10.3389/fimmu.2023.1264599 (PMC10755876; doi:10.3389/fimmu.2023.1264599)

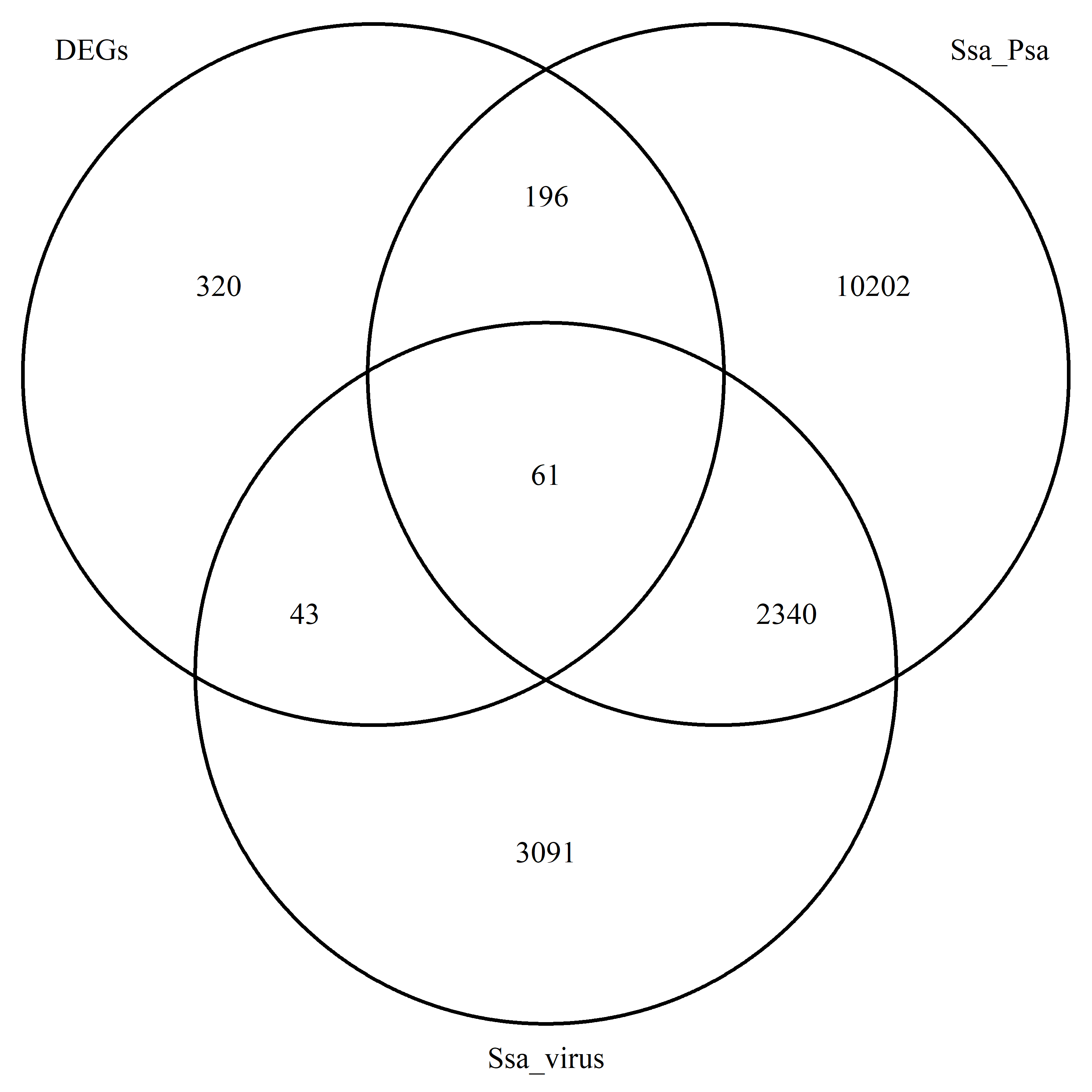

Supplement: Supplementary Figure 1 — Venn diagram of overlap between DEGs from different studies and ours. Venn diagram shows the overlap between our DEGs and the gene lists obtained from online data from studies about Atlantic salmon infected by P. salmonis and the virus. We grouped the DEGs by Ssa_Psa for the gene list from papers about Atlantic salmon infected by P. salmonis and Ssa_virus for the gene lists from papers about Atlantic salmon infected by virus (ISAv and POMV). [file Image_1.png]

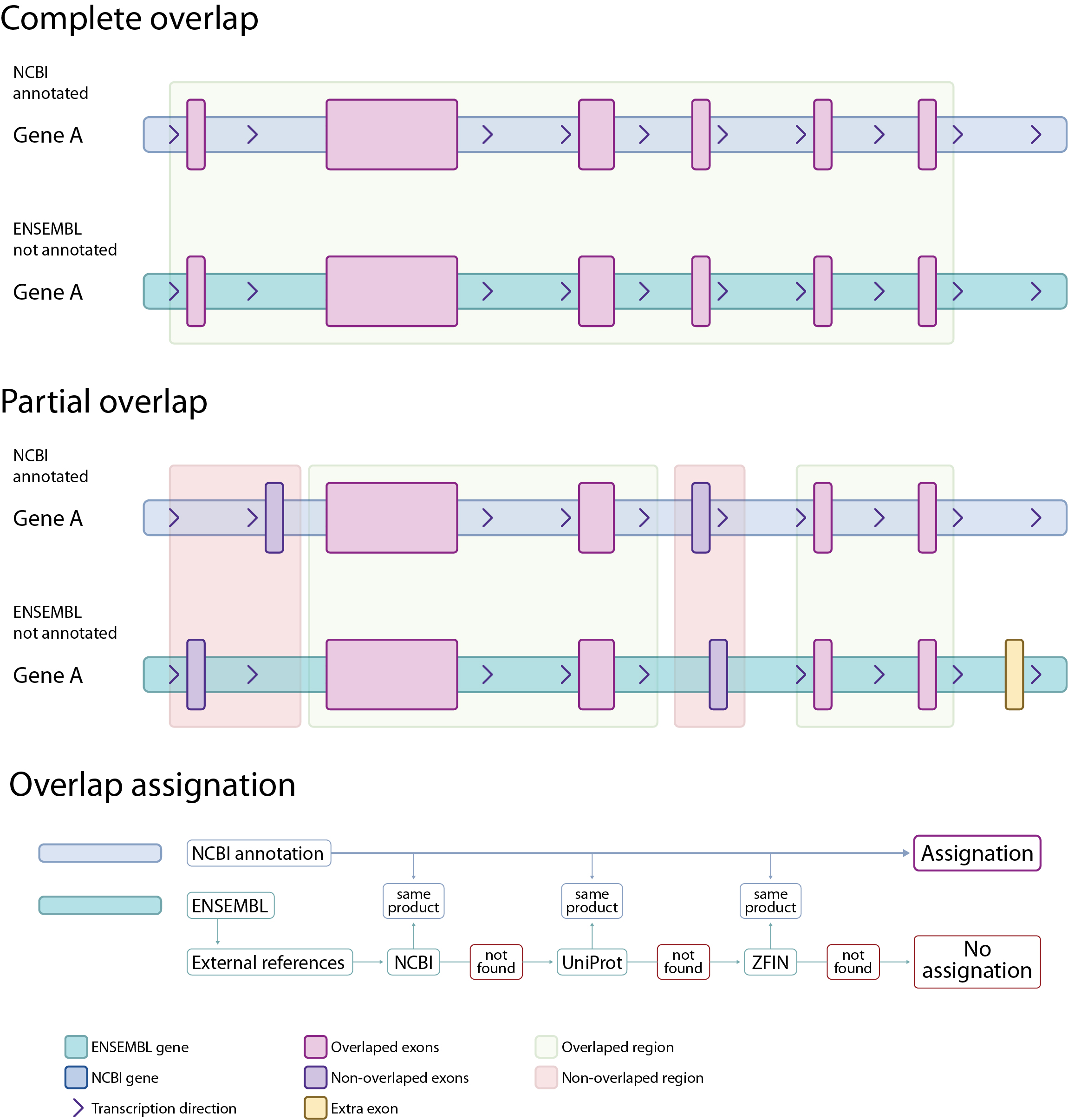

Supplement: SUPPLEMENTARY FIGURE 2 — Manual gene homologation from NCBI to ENSEMBL databases. Gene ID homologation was carried out by comparison of genomic coordinates in NCBI Genome Data Viewer. The top panel represents a complete overlap between the exons of a gene annotation from both databases. The mid panel shows an example of a partial overlap of exons, in which the observed difference of exons coordinates between both databases and the absence of exons from one database from another. The bottom panel shows the decision flow diagram that was used to assign the homologation of a partial overlap between the gene annotation from NCBI and ENSEMBL, with the priority of databases to assign the gene ID when ENSEMBL external references matched NCBI annotation. [file Image_2.jpeg]

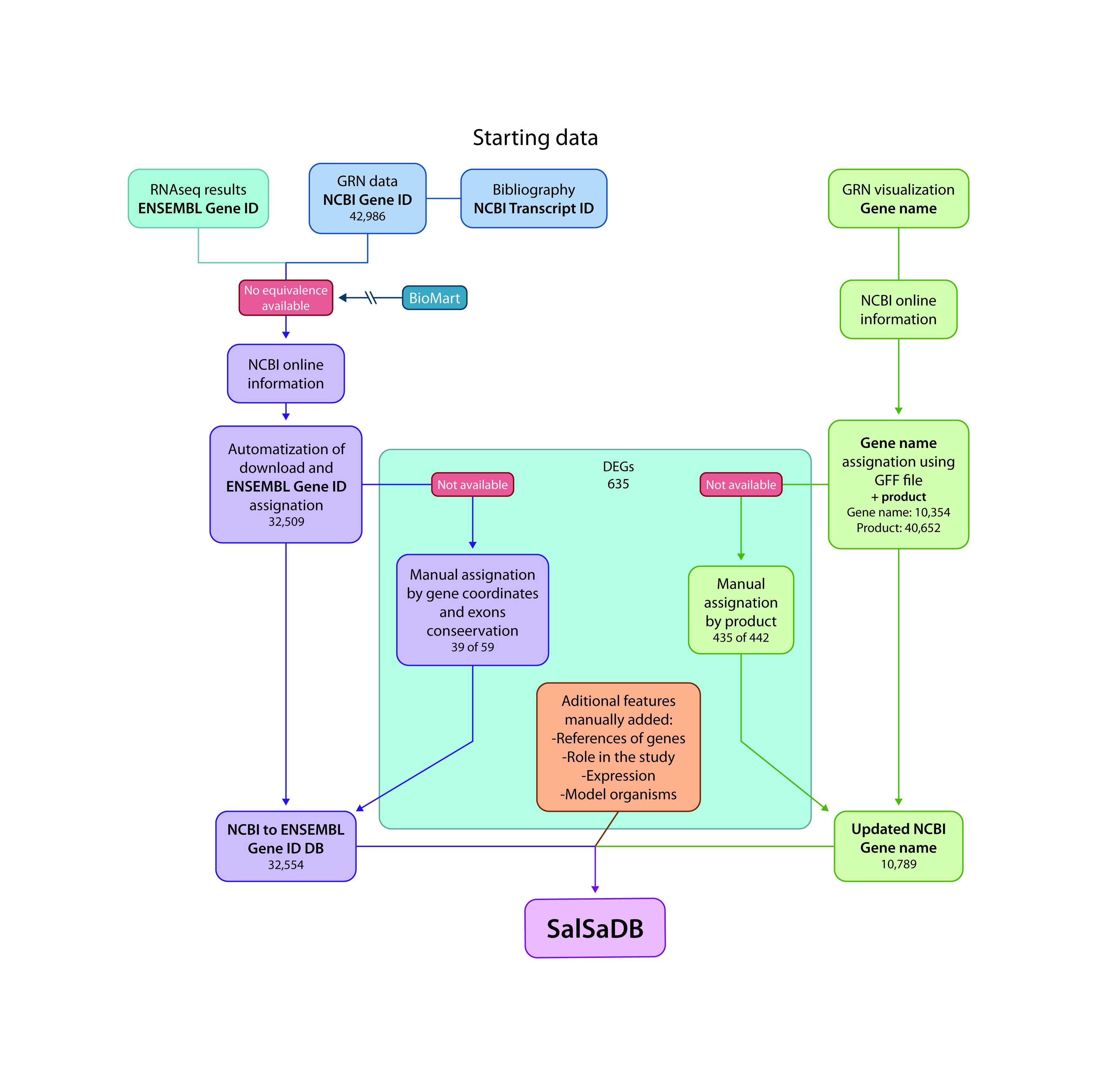

Supplement: SUPPLEMENTARY FIGURE 3 — SalSaDB recompilation diagram. SalSaDB was constructed using information from several databases and research papers to improve Atlantic salmon genomic information for this work. This diagram shows the quantity of genes successfully gathered and linked with NCBI information, with the steps we followed to improve the automatically obtained data for our DEGs. [file Image_3.jpeg]
