# Supplementary material for: KLF17 is an important regulatory component of the transcriptomic response of Atlantic salmon macrophages to Piscirickettsia salmonis infection
Source: Front Immunol. 2023 Dec 14;14:1264599. doi: 10.3389/fimmu.2023.1264599 (PMC10755876; doi:10.3389/fimmu.2023.1264599)
Supplement: Supplementary File 1 — rRNA sequences of Atlantic salmon.txt. [file DataSheet_1.zip › Supplementary File 1-2.docx]

**SUPPLEMENTARY FILE 1**

>AJ427629.1 Salmo salar 18S rRNA gene

TGGTTGATTCTGCCAGTAGCATATGCTTGTCTCAAAGATTAAGCCATGCAAGTCTAAGTACACACGGCCG

GTACAGTGAAACTGCGAATGGCTCATTAAATCAGTTATGGTTCCTTTGATCGCTCCAACGTTACTTGGAT

AACTGTGGCAATTCTAGAGCTAATACATGCAGACGAGCGCTGACCTCCGGGGATGCGTGCATTTATCAGA

CCCAAAACCCATGCGGGCCAATCTCGGTTGCCCCGGCCGCTTTGGTGACTCTAGATAACCTCGAGCCGAT

CGCGCGCCCTTTGTGGCGGTGACGTCTCATTCGAATGTCTGCCCTATCAACTTTCGATGGTACTTTCTGT

GCCTACCATGGTGACCACGGGTAACGGGGAATCAGGGTTCGATTCCGGAGAGGGAGCCTGAGAAACGGCT

ACCACATCCAAAGAAGGCAGCAGGCGCCCAAAATTACCACTCCCCACTCGGGGAGGTAGTGACCAAAAAT

AACAATACAGGACTCTTTCGAGGCCCCGTAATTGGAATGAGTACACTTTAAATCCTTTAACGAGGATCCA

TTGGAGGGCAAGTCTGGTGCCAGCAGCCGCGGTAATTCCAGCTCCAATAGCGTATCTTAAAGTTGCTGCA

GTTAAAAAGCTCGTAGTTGGATCTCGGGATCGAGCTGGCGGTCCGCCGCGAGGCGAGCTACCGCCTGTCC

CAGCCCCTGCCTCTCGGCGCCCCCTCGATGCTCTTAACTGAGTGTCCCGCGGGGTCCGAAGCGTTTACTT

TGAAAAAATTAGAGTGTTCAAAGCAGGCCCGGTCGCCTGAATACCGCAGCTAGGAATAATGGAATAGGAC

TCCGGTTCTATTTTGTGGGTTTTTCTTCTGAACTGGGGCCATGATTAAGAGGGACGGCCGGGGGCATTCG

TATTGTGCCGCTAGAGGTGAAATTCTTGGACCGGCGCAAGACGGACGAAAGCGAAAGCATTTGCCAAGAA

TGTTTTCATTAATCAAGAACGAAAGTCGGAGGTTCGAAGACGATCAGATACCGTCGTAGTTCCGACCATA

AACGATGCCAACTAGCGATCCGGCGGCGTTATTCCCATGACCCGCCGGGCAGCGTCCGGGAAACCAAAGT

CTTTGGGTTCCGGGGGGAGTATGGTTGCAAAGCTGAAACTTAAAGGAATTGACGGAAGGGCACCACCAGG

AGTGGAGCCTGCGGCTTAATTTGACTCAACACGGGAAACCTCACCCGGCCCGGACACGGAAAGGATTGAC

AGATTGATAGCTCTTTCTCGATTCTGTGGGTGGTGGTGCATGGCCGTTCTTAGTTGGTGGAGCGATTTGT

CTGGTTAATTCCGATAACGAACGAGACTCCGGCATGCTAACTAGTTATGCGGCCCCGAGCGGTCGGCGTC

CAACTTCTTAGAGGGACAAGTGGCGTTCAGCCACACGAGATTGAGCAATAACAGGTCTGTGATGCCCTTA

GATGTCCGGGGCTGCACGCGCGCCACACTGAGCGGATCAGCGTGTGTCTACCCTTCGCCGAGAGGCGTGG

GTAACCCGCTGAACCCCACTCGTGATAGGGATTGGGGATTGCAATTATTTCCCATGAACGAGGAATTCCC

AGTAAGCGCGGGTCATAAGCTCGCGTTGATTAAGTCCCTGCCCTTTGTACACACCGCCCGTCGCTACTAC

C

>Z18745.1 Salmo salar 28S ribosomal RNA (D8 domain)

GATCAAATTGGCAGCGGCGACTCTGGACGCGTGCCGCTTCTCGCGGATCTCCCCAGCTACGGTGCT

>Z18691.1 Salmo salar 28S rRNA, 5' end (C1, D1, and C2 domains)

AAAGATCGACCTCAGATCAGACGAGGCAACCCGCTGAATTTAAGCATATTACTAAGCGGAGGAAAAGAAA

CTACCGGATTCCCTCAGTAGCGGCGAGCGAAGAGGGAAGGGCCCAGCGCCGAATCCCTGTCCGACGGGCG

GGCATGGGAAATGTGGCGTATAGAAGACCGCTTTGCCCGGTGTCGATCGGGGGCCTGAGTCCTTCTGATC

GAGGCTAAACCCGTGGACGGTGTGAGGCCGGTAACGGCCCCCGTCGCGCCGGGGTCCGGTCTTCGAGTCG

GGTTGCTTGGGAATGCAGCCCAAAGTGGGTGGTAAACTCCATCTAAGGCTAAATACCGGCACGAGTCCGA

TAGTCGACAAGTACCGAAGGGAAAG

>Z18718.1 Salmo salar 28S ribosomal RNA (D2 domain)

GATCAAAGCACGGGGCCCTTTGCCCCCGGCGCGACTGTCAACCGGGTCGGACTGTCCTCAGTGCGCACCC

AACCGCGTTGCGTCGCCAGGGTAGGGATCGGCTCACGTAAACTGGCGCCAGGGGTCAGCGGCGATGTCGG

CAACCC

>EF417159.1 Salmo salar large subunit ribosomal RNA gene, partial sequence

GCCCAGCGCCGAATCCCTGTCCGACGGGCGGGCATGGGAAATGTGGCGTATAGAAGACCGCTTTGCCCGG

TGTCGATCGGGGGCCTGAGTCCTTCTGATCGAGGCTCAGCCCGTGGACGGTGTGAGGCCGGTAACGGCCC

CCGTCGCGCCGGGGTCCGGTCTTCTCGGAGTCGGGTTGCTTGGGAATGCAGCCCAAAGTGGGTGGTAAAC

TCCATCTAAGGCTAAATACCGGCACGAGTCCGATAGTCGACAAGTACCGTAAGGGAAAGTTGAAAAGAAC

TTTGAAGAGAGAGTTCAAGAGGGCGTGAAACCGTTGAGAGGTAAACGGGTGGGGTCCGCGCAGTCTGCCC

GGAGGATTCAACTCGGCGGGTCAGGGTCGGCCGTTCCGGTGTGGTCGGATCCCCTCGTGGGACTGACCCC

TGGTCGGGCTCGGCCCCCGCCGGGCGCATTTCCTCCGTCGGTGGTGCGCCGCGACCGGCTCTGGGTCGGC

TTGGAAGGGCTTGGGGCGAAGGTGGCTACCGGTTTCGGCCGTGAGCTTTACAGCGCTCCTGCTCCGTACT

CGCCGCTTTCCGGGGCCGAGGACTTAGTACCCGCTGCGTCATGTCCCCCTGCGGGGGGGCACGGGGCCCT

TTGCCCCCGGCGCGACTGTCAACCGGGTCGGACTGTCCTCAGTGCGCACCCAACCGCGTTGCGTCGCCAG

GGTAGGGATCGGCTCACGTAAACTGGCGCCAGGGGTCAGCGGCGATGTCGGCAACCCACCCGACCCGTCT

TGAAACACGGACCAAGGAGTCTAACGCACGCGCAAGTCAGAGGGTTTTCTCCGAACACACCCCGTGGCGC

AATGAAAGTGAGGGCCGGCGCGCGCCGGCTGAGGTGGGATCCCGGCCCTACGGGGTCGGGCGCACCACCG

GCCCGTCTCGCCCGCTCTGTCGGGGAGGTGGAGCGTGAGCGCGTGCGATAGGACCCGAAAGATGGTGAAC

TATGCCTGGGCAGGGCGAAGCCAGAGGAAACTCTGGTGGAGGTCCGTAGCGGTCCTGACGTGCAAATCGG

T

>KC984257.1 Salmo salar 16S ribosomal RNA gene, partial sequence; mitochondrial

TATGGAGCTTTAGACACCAGGCAGATCACGTCAAGTAACCTTGAATTAACAAGTAAAAACGCAGTGACCC

CTAGCCCATATGTCTTTGGTTGGGGCGACCGCGGGGGAAAACAAAGCCCCCATGTGGACTGGGGGCACTG

CCCCCACAACCAAGAGTCACAACTCTAAGTACCAGAATTTCTGACCAAAAATGATCCGGCATCACGCCGA

TCAACGGACCGAGTTACCCTA

>FJ620122.1 Salmo salar isolate 12S-U 12S ribosomal RNA gene, partial sequence; mitochondrial

TCTAGAACCGATAACCCCCGTTCAACCTCACCACCTCTTGTTTTCCCCGCCTATATACCACCGTCGTCAG

CTTACCCTGTGAAGGCCTTATAGTAAGCAAAATGGGCAAAACCCAA

>LC091619.1 Salmo salar mitochondrial gene for 12S rRNA, partial sequence, specimen_voucher: UW:021734

CACCGCGGTTATACGAGAGGCCCTAGTTGATAACTACCGGCGTAAAGAGTGGTTACGGAAAAATATTTAA

TAAAGCCGAACACCCCCTCAGCCGTCATACGCACCTGGGGGCACGAAGATCTACTACGAAAGCAGCTTTA

ATTATACCTGAACCCACGACAGCTACGACA

**SUPPLEMENTARY FILE 2**

>Illumina_Multiplexing_PCR_Primer_2.01_1

AACCCGTAGATCCGAACTTGTGAGATCGGAAGAGCACACGTCTGAACTCC

>Illumina_Multiplexing_PCR_Primer_2.01_2

TAGCTTATCAGACTGGTGTTGGCAGATCGGAAGAGCACACGTCTGAACTC

>Illumina_Multiplexing_PCR_Primer_2.01_3

TGAGAACTGAATTCCATAGATGGAGATCGGAAGAGCACACGTCTGAACTC

>Illumina_Multiplexing_PCR_Primer_2.01_4

AACCCGTAGATCCGAACTTGTAGATCGGAAGAGCACACGTCTGAACTCCA

>Illumina_Multiplexing_PCR_Primer_2.01_5

TGAGAACTGAATTCCATAGATGAGATCGGAAGAGCACACGTCTGAACTCC

>Illumina_Multiplexing_PCR_Primer_2.01_6

AAGCTGCCAGCTGAAGAACTGTAGATCGGAAGAGCACACGTCTGAACTCC

>Illumina_Multiplexing_PCR_Primer_2.01_7

AAGCTGCCAGCTGAAGAACTGAGATCGGAAGAGCACACGTCTGAACTCCA

>Illumina_Multiplexing_PCR_Primer_2.01_8

TAGCTTATCAGACTGGTGTTGGTAGATCGGAAGAGCACACGTCTGAACTC

>Illumina_Multiplexing_PCR_Primer_2.01_9

TAGCTTATCAGACTGGTGTTGGAGATCGGAAGAGCACACGTCTGAACTCC

>Illumina_Multiplexing_PCR_Primer_2.01_10

TGAGAACTGAATTCCATAGATGGTAGATCGGAAGAGCACACGTCTGAACT

>Illumina_Multiplexing_PCR_Primer_2.01_11

TAGCTTATCAGACTGGTGTTGACTAGATCGGAAGAGCACACGTCTGAACT

>Illumina_Multiplexing_PCR_Primer_2.01_12

TTCAAGTAATCCAGGATAGGCTAGATCGGAAGAGCACACGTCTGAACTCC

>TruSeq_Adapter_Index_1

AGATCGGAAGAGCACACGTCTGAACTCCAGTCACATCACGATCTCGTATG

>Illumina_DpnII_expression_Sequencing_Primer_

CACAAGTTCGGATCTACGGGTTGATCGTCGGACTGTAGAACTCTGAACGT

>Illumina_DpnII_expression_Sequencing_Primer_

GCCAACACCAGTCTGATAAGCTAGATCGTCGGACTGTAGAACTCTGAACG

>Illumina_DpnII_expression_Sequencing_Primer_

CCATCTATGGAATTCAGTTCTCAGATCGTCGGACTGTAGAACTCTGAACG

>Illumina_DpnII_expression_Sequencing_Primer_

ACAAGTTCGGATCTACGGGTTGATCGTCGGACTGTAGAACTCTGAACGTG

>Illumina_DpnII_expression_Sequencing_Primer_

CATCTATGGAATTCAGTTCTCAGATCGTCGGACTGTAGAACTCTGAACGT

>Illumina_DpnII_expression_Sequencing_Primer_

CAGTTCTTCAGCTGGCAGCTTGATCGTCGGACTGTAGAACTCTGAACGTG

>Illumina_DpnII_expression_Sequencing_Primer_

ACAGTTCTTCAGCTGGCAGCTTGATCGTCGGACTGTAGAACTCTGAACGT

>Illumina_DpnII_expression_Sequencing_Primer_

CCAACACCAGTCTGATAAGCTAGATCGTCGGACTGTAGAACTCTGAACGT

>Illumina_DpnII_expression_Sequencing_Primer_

ACCAACACCAGTCTGATAAGCTAGATCGTCGGACTGTAGAACTCTGAACG

>Illumina_DpnII_expression_Sequencing_Primer_

ACCATCTATGGAATTCAGTTCTCAGATCGTCGGACTGTAGAACTCTGAAC

>Illumina_DpnII_expression_Sequencing_Primer_

AGTCAACACCAGTCTGATAAGCTAGATCGTCGGACTGTAGAACTCTGAAC

>Illumina_DpnII_expression_Sequencing_Primer_

AGCCTATCCTGGATTACTTGAAGATCGTCGGACTGTAGAACTCTGAACGT

>Illumina_RNA_PCR_Primer

GATCGTCGGACTGTAGAACTCTGAACGTGTAGATCTCGGTGGTCGCCGTA

>NEBNext_Small_RNA_1

AGATCGGAAGAGCACACGTCTGAACTCCAGTCAC

>NEBNext_Small_RNA_2

GATCGTCGGACTGTAGAACTCTGAACGTGTAGATCTCGGTGGTCGCCGTATCATT

>Illumina_Single_End_Apapter_1

ACACTCTTTCCCTACACGACGCTGTTCCATCT

>Illumina_Single_End_Apapter_2

CAAGCAGAAGACGGCATACGAGCTCTTCCGATCT

>Illumina_Single_End_PCR_Primer_1

AATGATACGGCGACCACCGAGATCTACACTCTTTCCCTACACGACGCTCTTCCGATCT

>Illumina_Single_End_PCR_Primer_2

CAAGCAGAAGACGGCATACGAGCTCTTCCGATCT

>Illumina_Single_End_Sequencing_Primer

ACACTCTTTCCCTACACGACGCTCTTCCGATCT

>Illumina_Paired_End_Adapter_1

ACACTCTTTCCCTACACGACGCTCTTCCGATCT

>Illumina_Paired_End_Adapter_2

CTCGGCATTCCTGCTGAACCGCTCTTCCGATCT

>Illumina_Paried_End_PCR_Primer_1

AATGATACGGCGACCACCGAGATCTACACTCTTTCCCTACACGACGCTCTTCCGATCT

>Illumina_Paired_End_PCR_Primer_2

CAAGCAGAAGACGGCATACGAGATCGGTCTCGGCATTCCTGCTGAACCGCTCTTCCGATCT

>Illumina_Paried_End_Sequencing_Primer_1

ACACTCTTTCCCTACACGACGCTCTTCCGATCT

>Illumina_Paired_End_Sequencing_Primer_2

CGGTCTCGGCATTCCTACTGAACCGCTCTTCCGATCT

>Illumina_DpnII_expression_Adapter_1

ACAGGTTCAGAGTTCTACAGTCCGAC

>Illumina_DpnII_expression_Adapter_2

CAAGCAGAAGACGGCATACGA

>Illumina_DpnII_expression_PCR_Primer_1

CAAGCAGAAGACGGCATACGA

>Illumina_DpnII_expression_PCR_Primer_2

AATGATACGGCGACCACCGACAGGTTCAGAGTTCTACAGTCCGA

>Illumina_DpnII_expression_Sequencing_Primer

CGACAGGTTCAGAGTTCTACAGTCCGACGATC

>Illumina_NlaIII_expression_Adapter_1

ACAGGTTCAGAGTTCTACAGTCCGACATG

>Illumina_NlaIII_expression_Adapter_2

CAAGCAGAAGACGGCATACGA

>Illumina_NlaIII_expression_PCR_Primer_1

CAAGCAGAAGACGGCATACGA

>Illumina_NlaIII_expression_PCR_Primer_2

AATGATACGGCGACCACCGACAGGTTCAGAGTTCTACAGTCCGA

>Illumina_NlaIII_expression_Sequencing_Primer

CCGACAGGTTCAGAGTTCTACAGTCCGACATG

>Illumina_Small_RNA_Adapter_1

GTTCAGAGTTCTACAGTCCGACGATC

>Illumina_Small_RNA_Adapter_2

TCGTATGCCGTCTTCTGCTTGT

>Illumina_Small_RNA_RT_Primer

CAAGCAGAAGACGGCATACGA

>Illumina_Small_RNA_PCR_Primer_1

CAAGCAGAAGACGGCATACGA

>Illumina_Small_RNA_PCR_Primer_2

AATGATACGGCGACCACCGACAGGTTCAGAGTTCTACAGTCCGA

>Illumina_Small_RNA_Sequencing_Primer

CGACAGGTTCAGAGTTCTACAGTCCGACGATC

>Illumina_Multiplexing_Adapter_1

GATCGGAAGAGCACACGTCT

>Illumina_Multiplexing_Adapter_2

ACACTCTTTCCCTACACGACGCTCTTCCGATCT

>Illumina_Multiplexing_PCR_Primer_1.01

AATGATACGGCGACCACCGAGATCTACACTCTTTCCCTACACGACGCTCTTCCGATCT

>Illumina_Multiplexing_PCR_Primer_2.01

GTGACTGGAGTTCAGACGTGTGCTCTTCCGATCT

>Illumina_Multiplexing_Read1_Sequencing_Primer

ACACTCTTTCCCTACACGACGCTCTTCCGATCT

>Illumina_Multiplexing_Index_Sequencing_Primer

GATCGGAAGAGCACACGTCTGAACTCCAGTCAC

>Illumina_Multiplexing_Read2_Sequencing_Primer

GTGACTGGAGTTCAGACGTGTGCTCTTCCGATCT

>Illumina_PCR_Primer_Index_1

CAAGCAGAAGACGGCATACGAGATCGTGATGTGACTGGAGTTC

>Illumina_PCR_Primer_Index_2

CAAGCAGAAGACGGCATACGAGATACATCGGTGACTGGAGTTC

>Illumina_PCR_Primer_Index_3

CAAGCAGAAGACGGCATACGAGATGCCTAAGTGACTGGAGTTC

>Illumina_PCR_Primer_Index_4

CAAGCAGAAGACGGCATACGAGATTGGTCAGTGACTGGAGTTC

>Illumina_PCR_Primer_Index_5

CAAGCAGAAGACGGCATACGAGATCACTGTGTGACTGGAGTTC

>Illumina_PCR_Primer_Index_6

CAAGCAGAAGACGGCATACGAGATATTGGCGTGACTGGAGTTC

>Illumina_PCR_Primer_Index_7

CAAGCAGAAGACGGCATACGAGATGATCTGGTGACTGGAGTTC

>Illumina_PCR_Primer_Index_8

CAAGCAGAAGACGGCATACGAGATTCAAGTGTGACTGGAGTTC

>Illumina_PCR_Primer_Index_9

CAAGCAGAAGACGGCATACGAGATCTGATCGTGACTGGAGTTC

>Illumina_PCR_Primer_Index_10

CAAGCAGAAGACGGCATACGAGATAAGCTAGTGACTGGAGTTC

>Illumina_PCR_Primer_Index_11

CAAGCAGAAGACGGCATACGAGATGTAGCCGTGACTGGAGTTC

>Illumina_PCR_Primer_Index_12

CAAGCAGAAGACGGCATACGAGATTACAAGGTGACTGGAGTTC

>Illumina_DpnII_Gex_Adapter_1

GATCGTCGGACTGTAGAACTCTGAAC

>Illumina_DpnII_Gex_Adapter_1.01

ACAGGTTCAGAGTTCTACAGTCCGAC

>Illumina_DpnII_Gex_Adapter_2

CAAGCAGAAGACGGCATACGA

>Illumina_DpnII_Gex_Adapter_2.01

TCGTATGCCGTCTTCTGCTTG

>Illumina_DpnII_Gex_PCR_Primer_1

CAAGCAGAAGACGGCATACGA

>Illumina_DpnII_Gex_PCR_Primer_2

AATGATACGGCGACCACCGACAGGTTCAGAGTTCTACAGTCCGA

>Illumina_DpnII_Gex_Sequencing_Primer

CGACAGGTTCAGAGTTCTACAGTCCGACGATC

>Illumina_NlaIII_Gex_Adapter_1.01

TCGGACTGTAGAACTCTGAAC

>Illumina_NlaIII_Gex_Adapter_1.02

ACAGGTTCAGAGTTCTACAGTCCGACATG

>Illumina_NlaIII_Gex_Adapter_2.01

CAAGCAGAAGACGGCATACGA

>Illumina_NlaIII_Gex_Adapter_2.02

TCGTATGCCGTCTTCTGCTTG

>Illumina_NlaIII_Gex_PCR_Primer_1

CAAGCAGAAGACGGCATACGA

>Illumina_NlaIII_Gex_PCR_Primer_2

AATGATACGGCGACCACCGACAGGTTCAGAGTTCTACAGTCCGA

>Illumina_NlaIII_Gex_Sequencing_Primer

CCGACAGGTTCAGAGTTCTACAGTCCGACATG

>Illumina_Small_RNA_RT_Primer

CAAGCAGAAGACGGCATACGA

>Illumina_5p_RNA_Adapter

GTTCAGAGTTCTACAGTCCGACGATC

>Illumina_RNA_Adapter1

TCGTATGCCGTCTTCTGCTTGT

>Illumina_Small_RNA_3p_Adapter_1

ATCTCGTATGCCGTCTTCTGCTTG

>Illumina_Small_RNA_PCR_Primer_1

CAAGCAGAAGACGGCATACGA

>Illumina_Small_RNA_PCR_Primer_2

AATGATACGGCGACCACCGACAGGTTCAGAGTTCTACAGTCCGA

>Illumina_Small_RNA_Sequencing_Primer

CGACAGGTTCAGAGTTCTACAGTCCGACGATC

>TruSeq_Universal_Adapter

AATGATACGGCGACCACCGAGATCTACACTCTTTCCCTACACGACGCTCTTCCGATCT

>TruSeq_Adapter,_Index_1

GATCGGAAGAGCACACGTCTGAACTCCAGTCACATCACGATCTCGTATGCCGTCTTCTGCTTG

>TruSeq_Adapter,_Index_2

GATCGGAAGAGCACACGTCTGAACTCCAGTCACCGATGTATCTCGTATGCCGTCTTCTGCTTG

>TruSeq_Adapter,_Index_3

GATCGGAAGAGCACACGTCTGAACTCCAGTCACTTAGGCATCTCGTATGCCGTCTTCTGCTTG

>TruSeq_Adapter,_Index_4

GATCGGAAGAGCACACGTCTGAACTCCAGTCACTGACCAATCTCGTATGCCGTCTTCTGCTTG

>TruSeq_Adapter,_Index_5

GATCGGAAGAGCACACGTCTGAACTCCAGTCACACAGTGATCTCGTATGCCGTCTTCTGCTTG

>TruSeq_Adapter,_Index_6

GATCGGAAGAGCACACGTCTGAACTCCAGTCACGCCAATATCTCGTATGCCGTCTTCTGCTTG

>TruSeq_Adapter,_Index_7

GATCGGAAGAGCACACGTCTGAACTCCAGTCACCAGATCATCTCGTATGCCGTCTTCTGCTTG

>TruSeq_Adapter,_Index_8

GATCGGAAGAGCACACGTCTGAACTCCAGTCACACTTGAATCTCGTATGCCGTCTTCTGCTTG

>TruSeq_Adapter,_Index_9

GATCGGAAGAGCACACGTCTGAACTCCAGTCACGATCAGATCTCGTATGCCGTCTTCTGCTTG

>TruSeq_Adapter,_Index_10

GATCGGAAGAGCACACGTCTGAACTCCAGTCACTAGCTTATCTCGTATGCCGTCTTCTGCTTG

>TruSeq_Adapter,_Index_11

GATCGGAAGAGCACACGTCTGAACTCCAGTCACGGCTACATCTCGTATGCCGTCTTCTGCTTG

>TruSeq_Adapter,_Index_12

GATCGGAAGAGCACACGTCTGAACTCCAGTCACCTTGTAATCTCGTATGCCGTCTTCTGCTTG

>Illumina_RNA_RT_Primer

GCCTTGGCACCCGAGAATTCCA

>Illumina_RNA_PCR_Primer

AATGATACGGCGACCACCGAGATCTACACGTTCAGAGTTCTACAGTCCGA

>RNA_PCR_Primer,_Index_1

CAAGCAGAAGACGGCATACGAGATCGTGATGTGACTGGAGTTCCTTGGCACCCGAGAATTCCA

>RNA_PCR_Primer,_Index_2

CAAGCAGAAGACGGCATACGAGATACATCGGTGACTGGAGTTCCTTGGCACCCGAGAATTCCA

>RNA_PCR_Primer,_Index_3

CAAGCAGAAGACGGCATACGAGATGCCTAAGTGACTGGAGTTCCTTGGCACCCGAGAATTCCA

>RNA_PCR_Primer,_Index_4

CAAGCAGAAGACGGCATACGAGATTGGTCAGTGACTGGAGTTCCTTGGCACCCGAGAATTCCA

>RNA_PCR_Primer,_Index_5

CAAGCAGAAGACGGCATACGAGATCACTGTGTGACTGGAGTTCCTTGGCACCCGAGAATTCCA

>RNA_PCR_Primer,_Index_6

CAAGCAGAAGACGGCATACGAGATATTGGCGTGACTGGAGTTCCTTGGCACCCGAGAATTCCA

>RNA_PCR_Primer,_Index_7

CAAGCAGAAGACGGCATACGAGATGATCTGGTGACTGGAGTTCCTTGGCACCCGAGAATTCCA

>RNA_PCR_Primer,_Index_8

CAAGCAGAAGACGGCATACGAGATTCAAGTGTGACTGGAGTTCCTTGGCACCCGAGAATTCCA

>RNA_PCR_Primer,_Index_9

CAAGCAGAAGACGGCATACGAGATCTGATCGTGACTGGAGTTCCTTGGCACCCGAGAATTCCA

>RNA_PCR_Primer,_Index_10

CAAGCAGAAGACGGCATACGAGATAAGCTAGTGACTGGAGTTCCTTGGCACCCGAGAATTCCA

>RNA_PCR_Primer,_Index_11

CAAGCAGAAGACGGCATACGAGATGTAGCCGTGACTGGAGTTCCTTGGCACCCGAGAATTCCA

>RNA_PCR_Primer,_Index_12

CAAGCAGAAGACGGCATACGAGATTACAAGGTGACTGGAGTTCCTTGGCACCCGAGAATTCCA

>RNA_PCR_Primer,_Index_13

CAAGCAGAAGACGGCATACGAGATTTGACTGTGACTGGAGTTCCTTGGCACCCGAGAATTCCA

>RNA_PCR_Primer,_Index_14

CAAGCAGAAGACGGCATACGAGATGGAACTGTGACTGGAGTTCCTTGGCACCCGAGAATTCCA

>RNA_PCR_Primer,_Index_15

CAAGCAGAAGACGGCATACGAGATTGACATGTGACTGGAGTTCCTTGGCACCCGAGAATTCCA

>RNA_PCR_Primer,_Index_16

CAAGCAGAAGACGGCATACGAGATGGACGGGTGACTGGAGTTCCTTGGCACCCGAGAATTCCA

>RNA_PCR_Primer,_Index_17

CAAGCAGAAGACGGCATACGAGATCTCTACGTGACTGGAGTTCCTTGGCACCCGAGAATTCCA

>RNA_PCR_Primer,_Index_18

CAAGCAGAAGACGGCATACGAGATGCGGACGTGACTGGAGTTCCTTGGCACCCGAGAATTCCA

>RNA_PCR_Primer,_Index_19

CAAGCAGAAGACGGCATACGAGATTTTCACGTGACTGGAGTTCCTTGGCACCCGAGAATTCCA

>RNA_PCR_Primer,_Index_20

CAAGCAGAAGACGGCATACGAGATGGCCACGTGACTGGAGTTCCTTGGCACCCGAGAATTCCA

>RNA_PCR_Primer,_Index_21

CAAGCAGAAGACGGCATACGAGATCGAAACGTGACTGGAGTTCCTTGGCACCCGAGAATTCCA

>RNA_PCR_Primer,_Index_22

CAAGCAGAAGACGGCATACGAGATCGTACGGTGACTGGAGTTCCTTGGCACCCGAGAATTCCA

>RNA_PCR_Primer,_Index_23

CAAGCAGAAGACGGCATACGAGATCCACTCGTGACTGGAGTTCCTTGGCACCCGAGAATTCCA

>RNA_PCR_Primer,_Index_24

CAAGCAGAAGACGGCATACGAGATGCTACCGTGACTGGAGTTCCTTGGCACCCGAGAATTCCA

>RNA_PCR_Primer,_Index_25

CAAGCAGAAGACGGCATACGAGATATCAGTGTGACTGGAGTTCCTTGGCACCCGAGAATTCCA

>RNA_PCR_Primer,_Index_26

CAAGCAGAAGACGGCATACGAGATGCTCATGTGACTGGAGTTCCTTGGCACCCGAGAATTCCA

>RNA_PCR_Primer,_Index_27

CAAGCAGAAGACGGCATACGAGATAGGAATGTGACTGGAGTTCCTTGGCACCCGAGAATTCCA

>RNA_PCR_Primer,_Index_28

CAAGCAGAAGACGGCATACGAGATCTTTTGGTGACTGGAGTTCCTTGGCACCCGAGAATTCCA

>RNA_PCR_Primer,_Index_29

CAAGCAGAAGACGGCATACGAGATTAGTTGGTGACTGGAGTTCCTTGGCACCCGAGAATTCCA

>RNA_PCR_Primer,_Index_30

CAAGCAGAAGACGGCATACGAGATCCGGTGGTGACTGGAGTTCCTTGGCACCCGAGAATTCCA

>RNA_PCR_Primer,_Index_31

CAAGCAGAAGACGGCATACGAGATATCGTGGTGACTGGAGTTCCTTGGCACCCGAGAATTCCA

>RNA_PCR_Primer,_Index_32

CAAGCAGAAGACGGCATACGAGATTGAGTGGTGACTGGAGTTCCTTGGCACCCGAGAATTCCA

>RNA_PCR_Primer,_Index_33

CAAGCAGAAGACGGCATACGAGATCGCCTGGTGACTGGAGTTCCTTGGCACCCGAGAATTCCA

>RNA_PCR_Primer,_Index_34

CAAGCAGAAGACGGCATACGAGATGCCATGGTGACTGGAGTTCCTTGGCACCCGAGAATTCCA

>RNA_PCR_Primer,_Index_35

CAAGCAGAAGACGGCATACGAGATAAAATGGTGACTGGAGTTCCTTGGCACCCGAGAATTCCA

>RNA_PCR_Primer,_Index_36

CAAGCAGAAGACGGCATACGAGATTGTTGGGTGACTGGAGTTCCTTGGCACCCGAGAATTCCA

>RNA_PCR_Primer,_Index_37

CAAGCAGAAGACGGCATACGAGATATTCCGGTGACTGGAGTTCCTTGGCACCCGAGAATTCCA

>RNA_PCR_Primer,_Index_38

CAAGCAGAAGACGGCATACGAGATAGCTAGGTGACTGGAGTTCCTTGGCACCCGAGAATTCCA

>RNA_PCR_Primer,_Index_39

CAAGCAGAAGACGGCATACGAGATGTATAGGTGACTGGAGTTCCTTGGCACCCGAGAATTCCA

>RNA_PCR_Primer,_Index_40

CAAGCAGAAGACGGCATACGAGATTCTGAGGTGACTGGAGTTCCTTGGCACCCGAGAATTCCA

>RNA_PCR_Primer,_Index_41

CAAGCAGAAGACGGCATACGAGATGTCGTCGTGACTGGAGTTCCTTGGCACCCGAGAATTCCA

>RNA_PCR_Primer,_Index_42

CAAGCAGAAGACGGCATACGAGATCGATTAGTGACTGGAGTTCCTTGGCACCCGAGAATTCCA

>RNA_PCR_Primer,_Index_43

CAAGCAGAAGACGGCATACGAGATGCTGTAGTGACTGGAGTTCCTTGGCACCCGAGAATTCCA

>RNA_PCR_Primer,_Index_44

CAAGCAGAAGACGGCATACGAGATATTATAGTGACTGGAGTTCCTTGGCACCCGAGAATTCCA

>RNA_PCR_Primer,_Index_45

CAAGCAGAAGACGGCATACGAGATGAATGAGTGACTGGAGTTCCTTGGCACCCGAGAATTCCA

>RNA_PCR_Primer,_Index_46

CAAGCAGAAGACGGCATACGAGATTCGGGAGTGACTGGAGTTCCTTGGCACCCGAGAATTCCA

>RNA_PCR_Primer,_Index_47

CAAGCAGAAGACGGCATACGAGATCTTCGAGTGACTGGAGTTCCTTGGCACCCGAGAATTCCA

>RNA_PCR_Primer,_Index_48

CAAGCAGAAGACGGCATACGAGATTGCCGAGTGACTGGAGTTCCTTGGCACCCGAGAATTCCA

>ABI_Dynabead_EcoP_Oligo

CTGATCTAGAGGTACCGGATCCCAGCAGT

>ABI_Solid3_Adapter_A

CTGCCCCGGGTTCCTCATTCTCTCAGCAGCATG

>ABI_Solid3_Adapter_B

CCACTACGCCTCCGCTTTCCTCTCTATGGGCAGTCGGTGAT

>ABI_Solid3_5'_AMP_Primer

CCACTACGCCTCCGCTTTCCTCTCTATG

>ABI_Solid3_3'_AMP_Primer

CTGCCCCGGGTTCCTCATTCT

>ABI_Solid3_EF1_alpha_Sense_Primer

CATGTGTGTTGAGAGCTTC

>ABI_Solid3_EF1_alpha_Antisense_Primer

GAAAACCAAAGTGGTCCAC

>ABI_Solid3_GAPDH_Forward_Primer

TTAGCACCCCTGGCCAAGG

>ABI_Solid3_GAPDH_Reverse_Primer

CTTACTCCTTGGAGGCCATG
