# Supplementary material for: KLF17 is an important regulatory component of the transcriptomic response of Atlantic salmon macrophages to Piscirickettsia salmonis infection
Source: Front Immunol. 2023 Dec 14;14:1264599. doi: 10.3389/fimmu.2023.1264599 (PMC10755876; doi:10.3389/fimmu.2023.1264599)
Supplement: Supplementary file 5 [file DataSheet_2.docx]

**Supplemental Materials and Methods**

**Database creation: SalSaDB**

SalMotifDB is a publicly available database containing information about overrepresented TF binding sites in the promoter regions of genes in different salmonid species (Mulugeta et al., 2019). From SalMotifDB, we downloaded three Atlantic salmon datasets: a) information about the motifs that a TF can recognize, b) the motifs present in the promoter of each gene, and c) the description of each gene. With these three datasets, we were able to change the SalMotifDB gene ID for the NCBI gene ID (gene description dataset) to match the motifs present in the promoter of each gene with the motifs that a TF will recognize, obtaining the TF-target information. Finally, with this information, we obtained a reference GRN that was posteriorly analyzed using Gephi (Bastian et al., 2009), looking for the information about the total amount of nodes (each node represents a different gene), edges (every TF-target interaction), and the outdegree connectivity (the genes that are regulated by each TF). This reference GRN contains all possible TF-target interactions, meaning that it is not biased to a specific biological process, enabling its use to analyze the immune response in any biological context.

To date, only BioMart is available to link information between ENSEMBL and NCBI gene IDs; nevertheless, when acquiring that dataset, the number of equivalences was highly redundant, obtaining millions of IDs equivalences. Thus, the linkage of our transcriptomic information needed a different approach, as it was obtained with ENSEMBL gene IDs, with the GRN obtained using SalMotifDB datasets with NCBI gene IDs. To obtain NCBI to ENSEMBL gene ID equivalences to integrate our transcriptomic data (with ENSEMBL gene IDs) into the GRN obtained from SalMotifDB (NCBI gene IDs), we generated custom scripts for automatized downloading, reading, and registering the ENSEMBL gene ID associated with an NCBI gene ID from the gene summary file from NCBI webpage for each Atlantic salmon gene. The genes that did not show an ENSEMBL gene ID equivalent were assigned by comparing the genomic coordinates in NCBI Genome Data Viewer, which shows the location of both, ENSEMBL and NCBI genome annotation, and assigned the equivalent genes by exon conservation. We categorized the overlap between the two gene annotations (NCBI and ENSEMBL). These categories correspond to a complete overlap, which means that both the start site of transcription of the gene and the exons of the gene overlap with each other, and a partial overlap, in which most exons of the NCBI gene annotation overlap with the exons of the ENSEMBL gene annotation, and the start site of transcription could or not overlap. This information was supported by external references from ENSEMBL, which must be a conserved annotation with the NCBI gene annotation, we followed a hierarchy of databases from which we assigned gene annotation, allowing us to get the best quality annotation from external databases. The hierarchy consisted in selecting the more robust information, from NCBI, next UniProt, and finally ZFIN, and if there was no information in any of those databases, we did not assign any new annotation that allowed us to link ENSEMBL and NCBI to that gene (Supplementary Figure 2).

To complete our database, we added the gene symbol and the product of the gene. We obtained this information from the GFF file of the reference genome of Atlantic salmon (Ssal_v3.1). Additionally, to improve our analysis of DEGs, we manually curated those attributes, crossing information between different databases using the gene ID or gene product previously obtained from NCBI (NCBI, ENSEMBL, and UniProt) to achieve the best possible visualization and interpretation of our data (Supplementary Figure 3).

Finally, we included bibliography attributes that specify which genes were already found to be related to immune response, specifying the authors, the year, the model organisms used, and the DOI of the research article. As one of the standard identifiers used in microarray analysis, we added the transcripts IDs and accession IDs from NCBI to our database, as it will be helpful to integrate information generated similarly by other researchers.

With all this information, we were able to give depth to our database, allowing the user to know if there is information about genes and where to find it easily. Thus, we unified publicly accessible databases to create our small SalSaDB database, which will be publicly available on GitHub for public use (<https://github.com/SebastianReyesCerpa/SalSaDB>).

**References supplemental materials and methods.**

Mulugeta TD, Nome T, To T-H, Gundappa MK, Macqueen DJ, Våge DI, Sandve SR, Hvidsten TR. SalMotifDB: a tool for analyzing putative transcription factor binding sites in salmonid genomes. *BMC genomics* (2019) 20:1–8.

Bastian M, Heymann S, Jacomy M. Gephi: an open source software for exploring and manipulating networks. *Proceedings of the international AAAI conference on web and social media*. (2009). p. 361–362
