# Supplementary material for: KLF17 is an important regulatory component of the transcriptomic response of Atlantic salmon macrophages to Piscirickettsia salmonis infection
Source: Front Immunol. 2023 Dec 14;14:1264599. doi: 10.3389/fimmu.2023.1264599 (PMC10755876; doi:10.3389/fimmu.2023.1264599)
Supplement: Supplementary file 6 [file Table_1.docx]

Supplementary table 1. Transcriptomic analysis studies used to compare our results.

| Reference | Model | Times analyzed | Tissue | Transcriptomic analysis |
| --- | --- | --- | --- | --- |
| Moraleda et al. 2021 | *S. salar - P. salmonis* | 3, 9 dpi | HK, liver | RNAseq |
| Valenzuela-Miranda et al. 2018 | *S. salar - P. salmonis* | 3, 7, 14 dpi | HK, spleen | RNAseq |
| Xue et al. 2021 | *S. salar - P. salmonis* | 2, 7, 13, 21, 42 dpi | HK | Microarray - Agilent-025055 |
| Samsing et al. 2020 | *S. salar* - POMV | from 6 to 20 dpi | HK, spleen, liver | RNAseq |
| Gervais et al. 2023 | SHK1 - ISAV | 24, 48, 96 hpi | NA | scRNAseq |
| Kiron et al. 2020 | *S. salar* | NA | Intestine | RNAseq |
